# Clinical validation and utility of targeted nanopore sequencing for rapid pathogen diagnosis and precision therapy in lung cancer patients with pulmonary infections

**DOI:** 10.3389/fcimb.2025.1730098

**Published:** 2026-01-12

**Authors:** Qingmei Deng, Yanzhe Liu, Jian Zhang, Hongshan Zhang, Yiyong Zhang, Meng Wang, Min Jia, Dushan Ding, Yuqin Fang, Yunfei Wang, Hongcang Gu, Hongzhi Wang

**Affiliations:** 1 Science Island Branch of Graduate School, University of Science and Technology of China, Hefei, China; 2 Hefei Cancer Hospital of CAS, Institute of Health and Medical Technology, Hefei Institutes of Physical Science, Chinese Academy of Sciences, Hefei, China; 3 Hefei CAS-Hongshuo Medical Laboratory Ltd, Hefei, China; 4 Hangzhou Shengting Medical Technology Co., Ltd, Hangzhou, China; 5 Institute of Health Education, Hangzhou Center for Disease Control and Prevention, Hangzhou, China; 6 Institute of Health and Medical Technology, Hefei Institutes of Physical Science, Chinese Academy of Science, Hefei, China

**Keywords:** lung cancer, precision therapy, pulmonary infections, rapid pathogen diagnosis, targeted nanopore sequencing

## Abstract

**Background:**

Pulmonary infections are common in patients with lung cancer (LC), complicating diagnosis and treatment. This study explored the diagnostic performance and clinical utility of targeted nanopore sequencing (TNPseq) for detecting pathogens in LC-related pulmonary infections.

**Methods:**

A total of 143 patients with LC or benign pulmonary diseases complicated by pulmonary infections were included and stratified into diagnostic and therapeutic cohorts. Sputum samples underwent conventional culture, metagenomic next-generation sequencing (mNGS), and TNPseq analyses. Microbiota profiles were compared across disease groups and correlated with tumor therapy responses. In the therapeutic cohort, clinical outcomes were assessed between empirical therapy and TNPseq-guided therapy.

**Results:**

TNPseq identified a significantly higher proportion of clinically relevant pathogens compared to mNGS (48.76% vs. 16.80%, p < 0.001) and demonstrated superior sensitivity (81.25% vs. 68.75%), with a 40.7% reduction in turnaround time (16 hours vs. 27 hours). Both sequencing methods revealed an enrichment of *Lactobacillus* species in non-initial diagnosis lung cancer (NDLC) patients (p < 0.01). Patients exhibiting partial response or stable disease (PR/SD) showed increased abundance of *Neisseria*, *Veillonella*, and *Prevotella* species (p < 0.05). Clinical remission was achieved in all patients; however, 68.4% of those initially receiving empirical therapy subsequently required a switch to TNPseq-guided treatment due to its ineffectiveness. Compared to this empirical-to-TNPseq group, the median treatment duration was significantly shorter under direct TNPseq guidance (total: 6 days vs. 13 days, p < 0.01; LC subgroup: 5 days vs. 15.5 days, p < 0.05), thereby reducing unnecessary antibiotic exposure.

**Conclusions:**

By enabling rapid pathogen detection and profiling of the pulmonary microbiome, TNPseq facilitates targeted therapy and reduces antibiotic overuse in LC patients. These findings highlight the potential of TNPseq as a promising, rapid, and non-invasive diagnostic candidate for first-line use, offering a comprehensive view of both infection and host-microbe interactions in immunocompromised patients.

## Introduction

Infection represents the most common complication and the second leading cause of death among cancer patients, a population often immunocompromised due to disease and treatment (Nanayakkara et al., 2021). Lung cancer (LC), the second most common malignancy worldwide, poses a particularly high risk for respiratory infections, with more than half of patients developing pneumonia following anti-tumor treatment (Toi et al., 2018; Siegel et al., 2025; Wang et al., 2025). Prompt and accurate pathogen identification is essential for initiating effective antimicrobial therapy. However, traditional diagnostic methods are hampered by slow turnaround times, inadequate sensitivity, and a limited spectrum of detectable pathogens (Gadsby et al., 2016; Rajapaksha et al., 2019). Furthermore, invasive diagnostic procedures such as bronchoscopy and thoracentesis increase the risk of infection, especially hospital-acquired infection, in immunocompromised patients (Musso et al., 2025). These limitations highlight the urgent need for rapid, non-invasive diagnostic strategies specifically tailored for LC patients with suspected pulmonary infections.

Metagenomic next-generation sequencing (mNGS) has emerged as a powerful and comprehensive tool for pathogen detection, capable of identifying a broad spectrum of pathogen within 24–48 hours (Gu et al., 2021; Miller and Chiu, 2021). Nonetheless, its clinical adoption is constrained by relatively lengthy sequencing times, high computational demands, and persistent background interference from commensal microbiota (Govender et al., 2021). Third-generation sequencing methods, such as targeted nanopore sequencing (TNPseq), utilize specific primers or probes to selectively enrich pathogenic nucleic acids. This targeted approach effectively reduces background noise and enhances the detection of low-abundance pathogens, significantly accelerating diagnostic turnaround (Zhang et al., 2024; Chen et al., 2025). For cancer patients, analyzing sputum samples can avoid additional invasive procedures and associated risks. Previous research have demonstrated that mNGS exhibits superior pathogen detection capabilities compared to traditional culture methods in sputum samples (Mao et al., 2023). However, it remains unknown whether TNPseq can also demonstrate excellent performance compared to both mNGS and conventional methods in detecting pathogens among LC patients with respiratory infections.

Beyond its role in pathogen-related infections, the lung microbiome critically influences immune responses in LC, thereby potentially affecting therapeutic efficacy. Dysbiosis of the pulmonary microbiota can disrupt immune surveillance, promoting inflammatory responses through mechanisms such as Th17 cell recruitment and activation of the ERK/PI3K pathways in bronchial epithelial cells. Such disruptions may exacerbate inflammation, accelerate disease progression, and influence therapeutic outcomes (Wang et al., 2022). Previous research has identified distinct alterations in the sputum microbiota of non-small cell lung cancer (NSCLC) patients (Zeng et al., 2022), with *Veillonella parvula* notably associated with LC progression (Tsay et al., 2021). However, it remains unclear whether TNPseq-derived microbial profiles can offer insights into treatment outcomes.

To address these critical knowledge gaps, this study compares the diagnostic performance and microbial diversity identified by TNPseq and mNGS in LC patients and individuals with benign pulmonary diseases (BPD) presenting with suspected infections. Additionally, the study investigates the clinical outcomes associated with TNPseq-guided antimicrobial therapy. Through this comparative analysis, we aim to evaluate the potential of TNPseq as a first-line, non-invasive diagnostic tool for managing pulmonary infections in LC patients, ultimately improving the timeliness and precision of clinical interventions.

## Materials and methods

### Study design and patient information

This study retrospectively collected clinical data from 170 patients with suspected pulmonary infections who were treated at Hefei Cancer Hospital, Chinese Academy of Sciences, between January 2023 and December 2024. In the diagnostic cohort (n=120), sputum samples were tested in parallel by conventional culture, mNGS, and TNPseq for pathogen detection, while samples from the therapeutic cohort (n=50) underwent conventional culture and TNPseq testing. The inclusion criteria were: (1) clinical signs of pulmonary infection (fever, cough, sputum, dyspnea) or imaging abnormalities (pulmonary shadows, lesions); (2) available sputum samples for pathogen assays; and (3) complete clinical records. Exclusion criteria were: (1) non–LC tumors; (2) absent sputum samples; (3) hypersensitivity, known drug resistance, unresolved extrapulmonary infections, complex comorbidities, or poor sample quality. After excluding 26 non–LC patients and one without sputum, the final diagnostic cohort included 111 patients (63 LC and 48 BPD), and the therapeutic cohort included 32 patients (14 LC and 18 BPD) (**Supplementary Figure S1**).

This study was approved by the Medical Ethics Committee of the Hefei Cancer Hospital, Chinese Academy of Sciences (PJ-KY2023-092) and conducted in accordance with the principles of the Declaration of Helsinki (as revised in 2013) and relevant ethical and legal requirements. The requirement for written informed consent was waived because the study involved a retrospective analysis of residual specimens from standard clinical care.

### Sputum sample collection

Patients fasted for ≥1 hour prior to sample collection. Fresh expectorated sputum was aseptically transferred into sterile tubes (Feigelman et al., 2017). A qualified lower respiratory sputum specimen meets the following criteria: Squamous epithelial cells ≤10/Low Power Field (LPF) and Leukocytes ≥25/LPF. Culture specimens were processed within 30 minutes in the institutional microbiology lab; sequencing aliquots were stored at −80 °C and shipped to Hangzhou Shengting Medical Technology Co., Ltd. Both sites followed national pathogen-detection protocols.

### Culture

Standard culture techniques were employed to identify potential pathogens. Sputum specimens were inoculated onto selective media (Babio Biotechnology, Jinan, China) under aseptic conditions: blood agar, MacConkey agar, and chocolate agar for bacterial culture; sabouraud dextrose agar for fungal culture. Bacterial cultures were incubated at 36.5 ± 0.5 °C with 5% CO_2_ for 18–72 hours, whereas fungal cultures were incubated at 28.0 ± 0.5 °C for up to 7 days. Pathogen identification was performed with the VITEK 2 Compact system. Viral cultures were not conducted due to biosafety constraints.

### DNA extraction and sequencing

Nucleic acids extracted from sputum samples were used as templates. Sputum samples were processed according to the following protocol: 400μL of each sputum sample was liquefied with 2% NaOH, centrifuged, then washed twice with PBS before resuspension in lysis buffer. Mechanical lysis was performed with grinding beads, followed by enzymatic digestion. DNA was extracted using the QIAamp DNA Microbiome Kit (QIAGEN), purified with magnetic beads, and eluted in nuclease-free water. The extracted DNA was quantified using a Qubit 4 fluorometer (Thermo Fisher Scientific, Massachusetts, USA). No-template controls (NTCs) with blank elution buffer were run in parallel.

For mNGS, libraries were prepared using the KAPA EVOplus Kit (Roche, Pleasanton, CA, USA) following the manufacturer’s instructions. The amplification was carried out on an ABI 2720 thermocycler with the following conditions: initial denaturation at 98 °C for 45 s, followed by 9 cycles of 98 °C for 15 s, 60 °C for 30 s, 72 °C for 30 s, and a final extension at 72 °C for 1 min. Libraries were quantified with the Qubit 4 Fluorometer (Thermo Fisher Scientific, Waltham, MA, USA) using the Qubit 1×dsDNA HS Assay Kit, and sequenced on the NovaSeq 6000 System (Illumina, San Diego, CA, USA) with a 2×150 bp paired-end protocol.

For TNPseq, PCR amplification targeting the 16S rRNA gene of bacteria, the 18S rRNA genes and ITS region of fungi, specific viral fragments (e.g., SARS-CoV-2 ORF1ab), and drug resistance genes was conducted using an ABI 2720 thermocycler. The amplification was carried out with the following conditions: initial denaturation at 95 °C for 3 min, followed by 30 cycles of 95 °C for 30 s, 62 °C for 60 s, 72 °C for 60 s, and a final extension at 72 °C for 3 min. Purified PCR products were quantified and prepared for library preparation using the PCR Barcode Expansion Pack 1-96 (EXP-PBC096, Oxford Nanopore Technologies, Oxford, UK). Sequencing was carried out on a GridION platform (Oxford Nanopore Technologies, Oxford, UK), followed by barcode demultiplexing with Porechop, short-read filtering via NanoFilt (2.7.1), and taxonomic classification of high-quality reads through alignment against pathogen reference databases using NCBI BLAST after NTCs filtering. The coverage includes 28,699 microbial species such as bacteria, fungi, viruses, and atypical pathogens, along with the detection of over 100 common antibiotic resistance genes and macrolide drug resistance genes based on SNP point mutations.

### Filtering of sequencing results

Contaminants were identified by comparison with blank controls, including the sequencing blank, ward environmental, and biosafety cabinet environmental controls. Subsequently, any contaminating organisms detected in the samples were filtered out following established protocols (Langelier et al., 2018; Li et al., 2021). Different pathogen identification thresholds are established based on the distinct principles and data characteristics of the two sequencing technologies: for mNGS data, ≥5 reads for fungi and bacteria, and ≥1 read for viruses and atypical pathogens (e.g., *Mycoplasma Pneumonia*e, *Mycobacterium*, *Nocardia*, and *Aspergillus*); for TNPseq data, ≥3 reads for bacteria and ≥1 read for other pathogens (including *Mycobacterium* and *Nocardia*).

Sequences derived from protozoa and parasites identified in sputum samples were systematically excluded. Filtered reads were aligned to the human reference genome (GRCh38/hg38) for host DNA removal. The remaining reads were subsequently aligned against relevant pathogenic sequences in NCBI-BLAST to analyze potential pathogens present in the sample.

To distinguish between colonizing microorganisms and pathogens, we utilized established pathogenic risk detection thresholds, considering read counts, relative abundance, frequency of occurrence, and relevant patient physiological indices (Maddi et al., 2019; Chen et al., 2022). We also referred to the *Manual of Clinical Microbiology* (11th edition) to determine pathogenicity and classification level of the pathogens.

### Comparison of detected microorganisms with known microorganisms

A literature review was conducted to select 62 microorganisms (excluding RNA viruses) that are commonly detected in pulmonary infections of lung cancer patients (**Supplementary Table S1**) (Gazzoni et al., 2014; Maddi et al., 2019; Guo et al., 2021; Drokow et al., 2023).

### Clinical characterization and patient classification

Patients were classified based on their clinical diagnosis, which was primarily determined using microbiological examination, bronchoscopy, and radiography. Patients were categorized into LC and BPD groups. Within the LC group, patients were further classified into initial diagnosis lung cancer (IDLC) or non-initial diagnosis lung cancer (NDLC), depending on whether their LC diagnosis was newly established at our institution. Treatment responses were evaluated and classified according to the Response Evaluation Criteria in Solid Tumors (RECIST) as partial response (PR), stable disease (SD), or progressive disease (PD). Based on antibiotic treatment strategy, patients were stratified into either an empirical therapy group or a TNPseq-guided therapy group.

### Evaluation of detection efficiency

To evaluate diagnostic efficacy, sequencing results were compared against traditional diagnostic standards, including microbial culture and composite reference standards (CRS).

CRS status was independently adjudicated by two senior specialists: a chief physician in respiratory and critical care medicine and a chief technologist in clinical microbiology. They reviewed all conventional diagnostic data, including medical records, imaging, culture reports, serology/pathology, and treatment response. If the initial adjudications regarding a case’s infection status were discordant, a consensus discussion was initiated, strictly following current clinical guidelines. If disagreement persisted, a third senior clinical expert, who did not participate in the initial adjudication, was invited to serve as an arbitrator and make the final determination. A case was defined as CRS-positive (microbiologically confirmed infection) if it met the clinical diagnosis of pulmonary infection, satisfied ≥1 of the following criteria, with documented clinical improvement following targeted therapy (De Pauw et al., 2008; Rotstein et al., 2008): (1) Pathogenic bacteria are cultured from qualified lower respiratory tract secretions, protected specimen brushes via bronchoscopy, bronchoalveolar lavage fluid, lung tissue, or sterile body fluids, and the findings are consistent with the clinical presentation; (2) Fungal elements are observed through histopathology, cytopathology, or direct microscopy of lung tissue specimens, accompanied by relevant evidence of tissue damage; (3) Seroconversion from negative to positive for specific IgM antibodies against atypical pathogens or viruses, or a fourfold or greater increase in specific IgG antibody titers between acute and convalescent paired serum samples. Additionally, we provide an illustrative case of a patient who was culture-negative but was adjudicated as having *Mycoplasma Pneumoniae* infection based on CRS criteria (**Supplementary Figure S2**).

### Bioinformatic analysis

The α-diversity of microorganisms was evaluated using the Shannon index, ACE, Chao1, Richness, and Simpson index. β-diversity was analyzed using unweighted UniFrac, weighted UniFrac, Jaccard, and Bray-Curtis distances computed with QIIME2, and visualized using principal coordinate analysis (PCoA). Comparative pathogen composition across taxonomic levels was statistically analyzed using Statistical Analysis of Metagenomic Profiles (STAMP, v2.1.3) and the linear discriminant analysis effect size (LEfSe) method.

### Statistical analysis

Statistical analyses were conducted using SPSS 17.0 (IBM, USA), GraphPad Prism 5.0 (GraphPad Software, USA), and R Studio 4.3.0. Continuous variables were reported as median with interquartile range (IQR). Categorical variables were summarized as frequencies with percentages. Comparisons between two groups utilized Student’s t-test for normally distributed variables and the Mann-Whitney U test for non-normally distributed variables. Categorical data were analyzed using Fisher’s exact test or chi-square test as appropriate. Differences among multiple groups were assessed using one-way ANOVA. A two-tailed p-value <0.05 was considered statistically significant.

## Results

### Study cohorts and baseline characteristics

A total of 143 patients were included, divided into diagnostic (n=111) and treatment (n=32) cohorts (**Table 1**). The diagnostic cohort had a median age of 66 years (IQR: 55.5-72), comprising 70.27% males, with LC present in 56.76%. Common comorbidities included hypertension (18.92%), diabetes (7.21%), and cardiopathy (6.31%). The primary clinical symptoms were cough (34.23%) and expectoration (27.03%). The treatment cohort had a median age of 70 years (IQR: 61-73.5), 68.75% were male, and LC was diagnosed in 43.75% of patients. Hypertension (18.75%) and diabetes (15.63%) were common. Predominant symptoms included cough (59.38%) and expectoration (56.25%).

**Table 1:** Clinical characteristics of the participants.

Characteristic (median (IQRs) or no. (%))	Diagnostic cohort (n=111)	Treatment cohort (n=32)	P value
Age (Years)	66 (55.5, 72)	70 (61, 73.5)	0.0915
Gender			
Male	78 (70.27%)	22 (68.75%)	>0.9999
Female	33 (29.37%)	10 (31.25%)	
Lung diseases			
Lung cancer	63 (56.76%)	14 (43.75%)	0.2294
Benign pulmonary disease	48 (43.24%)	18 (56.25%)	
Comorbidity			
Hypertension	21 (18.92%)	6 (18.75%)	>0.9999
Diabetes	8 (7.21%)	5 (15.63%)	0.166
Cardiopathy	7 (6.31%)	0	0.3492
Hyperlipidemia	2 (1.80%)	1 (3.12%)	0.5352
Clinical symptoms			
Fever	20 (18.02%)	12 (37.50%)	0.0293
Cough	38 (34.23%)	19 (59.38%)	0.0139
Expectoration	30 (27.03%)	18 (56.25%)	0.0029
Chest distress	19 (17.12%)	12 (37.50%)	0.0261
Asthma	8 (7.21%)	3 (9.38%)	0.7097
Hemoptysis	6 (5.41%)	5 (15.63%)	0.0689

### TNPseq demonstrates superior operational efficiency and higher clinical relevance in pathogen identification

**Figure 1:**
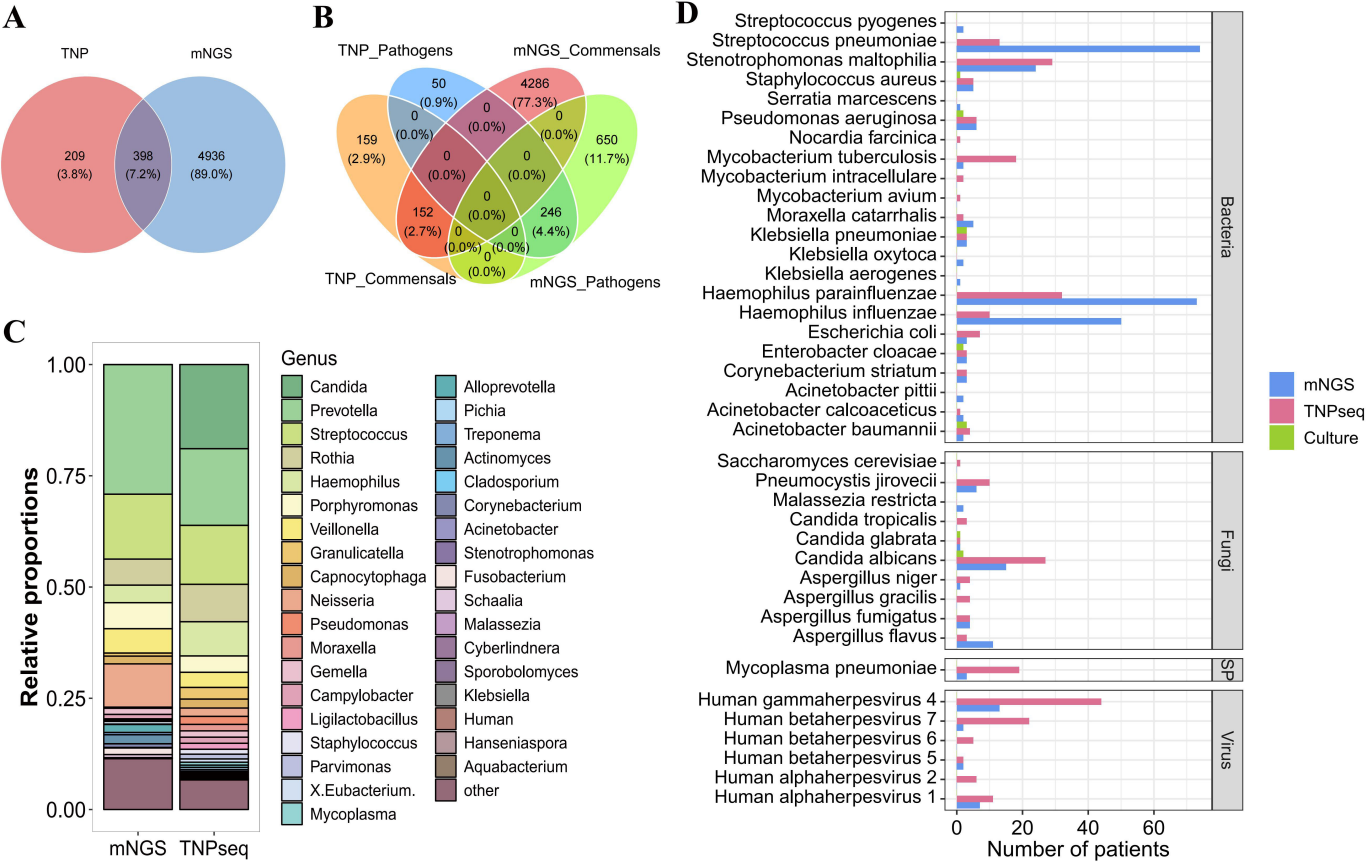
Comparison of pathogen detection performance between TNPseq and mNGS. **(A)** Microbial species number obtained by mNGS and TNPseq; **(B)** Comparative Venn diagram of pathogenicity classification for microorganisms detected via mNGS versus TNPseq; Commensals: microbes that typically colonize the host without causing disease under normal conditions; **(C)** Comparison of relative abundance at the genus level between mNGS and TNPseq; **(D)** Detection of common lung cancer-associated pathogens by mNGS, TNPseq, and culture. mNGS, Metagenomic next-generation sequencing; TNPseq, Targeted nanopore sequencing; SP, Specific pathogen.

To evaluate the diagnostic performance of TNPseq, we first compared it with mNGS and conventional culture. Despite generating a lower total volume of sequencing data, especially for bacteria (**Figures 1A**, **S3**), TNPseq exhibited a decisive advantage in clinical applicability. It identified a significantly higher proportion of clinically relevant potential pathogens (48.76%, 296/607) compared to mNGS (16.80%, 896/5,334; P<0.001; **Figure 1B**).

Operationally, TNPseq achieved a 40.7% faster turnaround (16 hours for TNPseq vs. 27 hours for mNGS), facilitated by rapid real-time sequencing (7 hours for TNPseq vs. 22 hours for mNGS; **Supplementary Table S2**).

Culture tests were performed on all samples, with 11 yielding positive results. Both sequencing methods demonstrated 72.73% sensitivity for culture-positive samples. Within the cohort of 16 CRS-positive cases, TNPseq showed higher sensitivity (81.25%) than mNGS (68.75%; **Supplementary Table S3**), highlighting TNPseq’s strength in rapidly and precisely identifying clinically relevant pathogens. Additionally, TNPseq detected a slightly higher number of pathogens compared to mNGS (**Supplementary Table S4**).

Despite the difference in data volume, the two sequencing methods showed strong agreement in detecting common and critical respiratory pathogens. Both methods identified common microorganisms including *Prevotella*, *Streptococcus*, and *Haemophilus* (**Figure 1C**). Concordance between methods was high for lung cancer-associated pathogens such as *Haemophilus influenzae*, S*treptococcus pneumoniae*, and *human gammaherpesvirus 4* (**Figure 1D**). This high concordance confirms that TNPseq retains the accurate detection capability of broad-spectrum sequencing while offering enhanced speed and specificity.

### TNPseq could reveal divergent pulmonary microbiomes across disease groups

**Figure 2:**
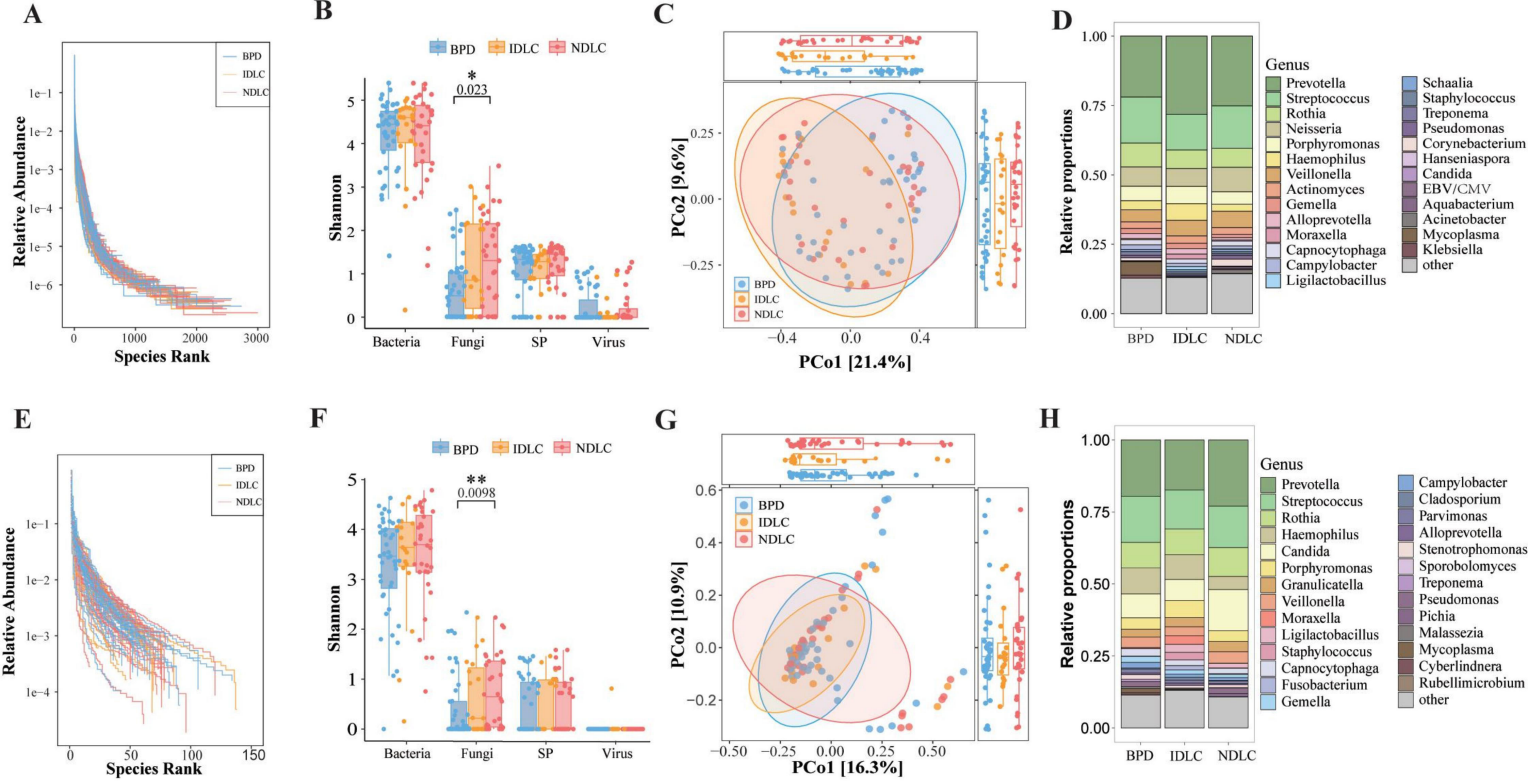
Comparison of mNGS and TNPseq for pathogen detection in BPD, IDLC, and NDLC cohorts. **(A)** RACs demonstrate that mNGS achieves greater taxonomic breadth and evenness, evidenced by longer, flatter curves; **(B)** α-diversity (via Shannon index) was calculated using mNGS-detected data for assay comparison; **(C)** β-diversity was calculated via PCoA using mNGS-detected data for assay comparison; **(D)** Relative abundance of each pathogen in each group, as measured by mNGS; **(E)** RACs demonstrate that TNPseq more reliably detects dominant pathogens, as reflected by steeper curves. **(F)** α-diversity was calculated using TNPseq-detected data for assay comparison; **(G)** β-diversity was calculated using TNPseq-detected data for assay comparison; **(H)** Relative abundance of each pathogen in each group, as measured by TNPseq. BPD: Benign pulmonary disease; IDLC, Initial diagnosis lung cancer; NDLC, Non-initial diagnosis lung cancer; RACs, Rank abundance curves; PCoA, Principal coordinates analysis. P-values determined by Wilcoxon rank sum test. *P<0.05, **P<0.01; SP, Specific pathogen.

We examined microbial diversity and composition across patients with BPD, IDLC, and NDLC. Rank abundance curves (RACs) differed by sequencing method: mNGS showed broader taxonomic diversity and evenness, whereas TNPseq had steeper slopes, indicating dominance by fewer pathogens (**Figures 2A, E**). Despite these methodological differences, both approaches exhibited consistent trends. Significant differences in fungal α-diversity emerged between BPD and NDLC groups using both mNGS (P = 0.023) and TNPseq (P = 0.0098), as assessed by the Shannon index (**Figures 2B, F**). However, no significant differences were observed in the ACE, Chao1, Richness, or Simpson indices(**Supplementary Figure S4**). Furthermore, β-diversity did not differ significantly among the three groups (**Figures 2C, G**). Taxon abundance rankings aligned closely between methods, with *Prevotella*, *Streptococcu*s, and *Rothia* consistently dominating across all patient groups (**Figures 2D, H**).

**Figure 3:**
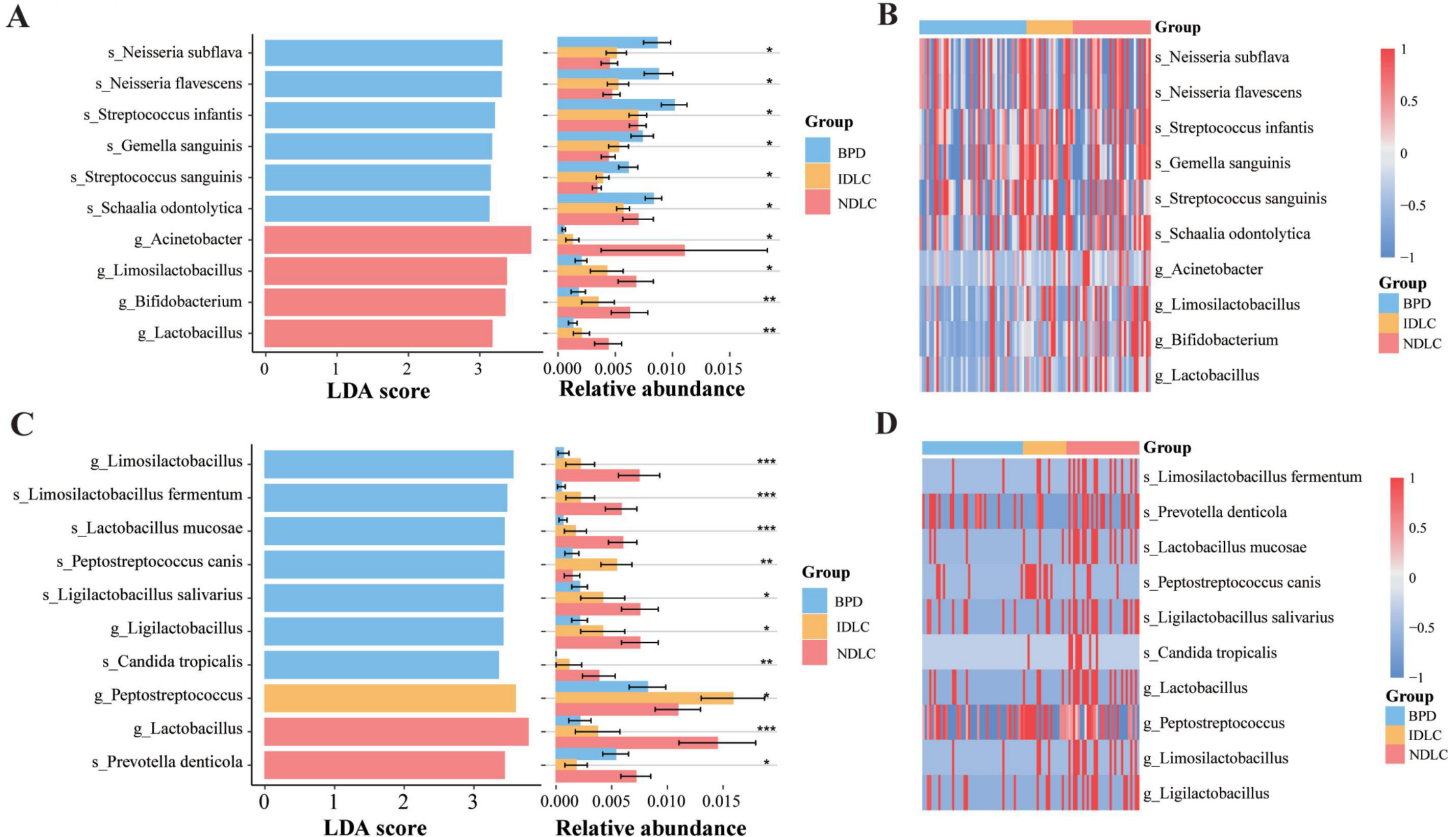
LEfSe results comparing pathogens among BPD, IDLC, and NDLC. **(A)** LDA scores and relative abundance jointly highlighted mNGS-derived biomarkers across three clinical groups: BPD, IDLC, and NDLC; **(B)** Heatmaps show mNGS-detected pathogen distribution across samples in the BPD, IDLC, and NDLC groups; **(C)** LDA scores and relative abundance jointly highlighted TNPseq-derived biomarkers across three clinical groups: BPD, IDLC, and NDLC; **(D)** Heatmaps show TNPseq-detected pathogen distribution across samples in the BPD, IDLC, and NDLC groups. LEfSe, Linear discriminant analysis effect size; LDA, Linear discriminant analysis. *P<0.05, **P<0.01; ***P<0.001.

LEfSe analysis identified distinct microbial biomarkers associated with specific disease groups. Both sequencing platforms detected significant enrichment of *Lactobacillus* in NDLC patients (mNGS: P<0.01; TNPseq: P<0.001) (**Figures 3A, C**), which was further confirmed through heatmap visualization (**Figures 3B, D**).

### TNPseq may uncover differential microbiome features linked to tumor treatment response

To investigate the microbiome differences between the tumor response group (PR/SD) and the progression group (PD), we assessed microbial diversity and composition in these two groups. RAC revealed that mNGS detected greater taxonomic breadth and evenness, whereas TNPseq exhibited steeper slopes, indicating the predominance of dominant pathogens (**Supplementary Figures S5A, E**). Both platforms demonstrated no significant differences in overall α-diversity (**Supplementary Figures S5B, F**, **S6**) or β-diversity (**Supplementary Figures S5C, G**). The concordance between methods was further evidenced by taxon abundance rankings, which identified *Prevotella* and *Streptococcus* as the dominant genera in both groups (**Supplementary Figures S5D, H**).

**Figure 4:**
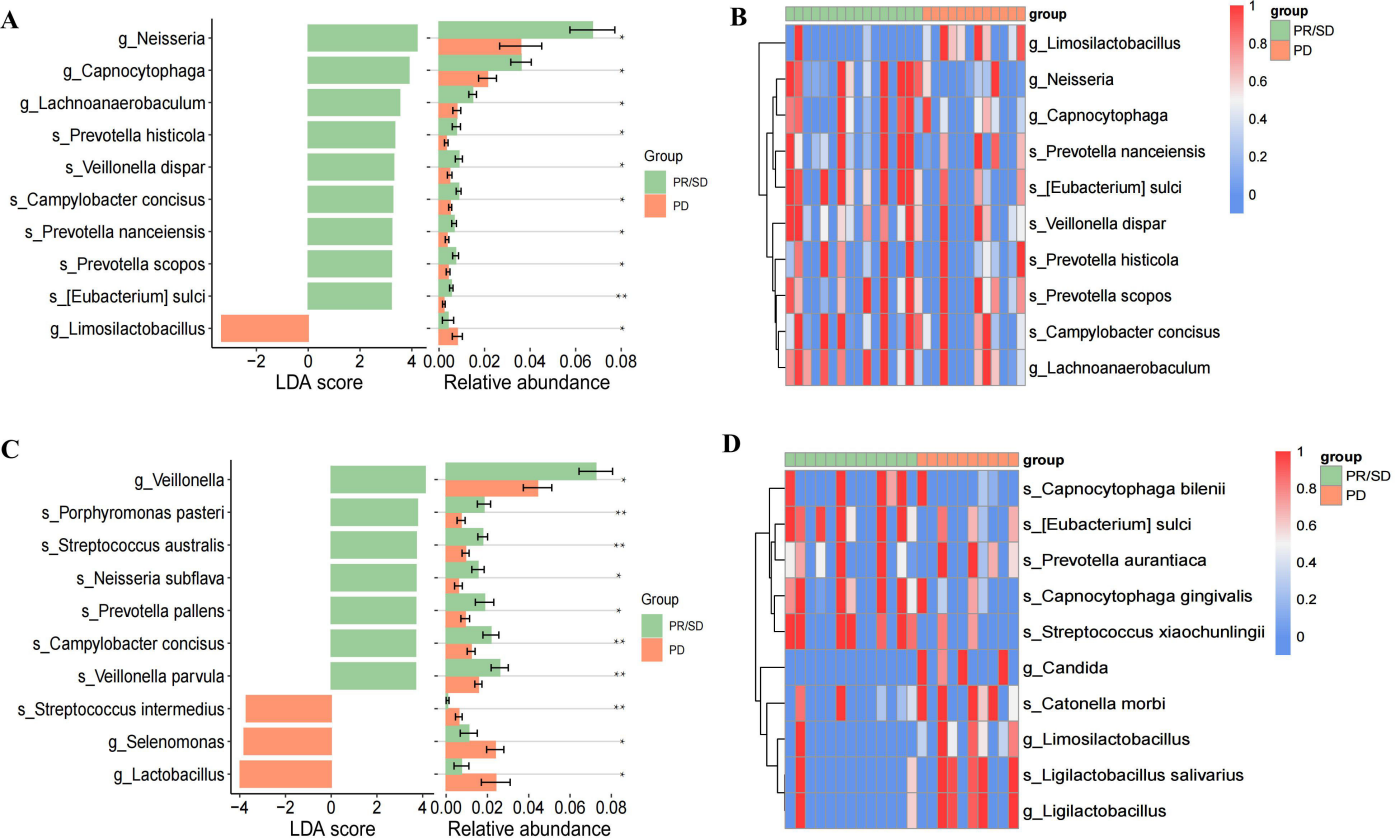
LEfSe results comparing pathogens between PR/SD and PD groups. **(A)** LDA scores coupled with relative abundance revealed mNGS-specific biomarkers differentiating PR/SD from PD; **(B)** Heatmaps visualize mNGS-based pathogen distribution across PR/SD and PD cohorts; **(C)** LDA scores coupled with relative abundance revealed TNPseq-specific biomarkers differentiating PR/SD from PD; **(D)** Heatmaps visualize TNPseq-based pathogen distribution across PR/SD and PD cohorts. PR, partial response; SD, stable disease; PD, Progressive disease. *P<0.05, **P<0.01.

Given the limited sample size, LEfSe analysis was conducted as an exploratory investigation into bacterial species differences. The results suggested that *Neisseria*, *Veillonella*, and *Prevotella* may be associated with the PR/SD group, showing significant enrichment (P < 0.05) by both methods (**Figures 4A, C**). Visualization using heatmaps confirmed their relatively elevated abundance in PR/SD (**Figures 4B, D**). Additionally, LEfSe analysis of TNPseq data also revealed significant enrichment of *Streptococcus intermedius* in PD group (P < 0.01) (**Figure 4C**).

### TNPseq-guided therapy significantly shortens antibiotic treatment duration and improves outcomes

**Figure 5:**
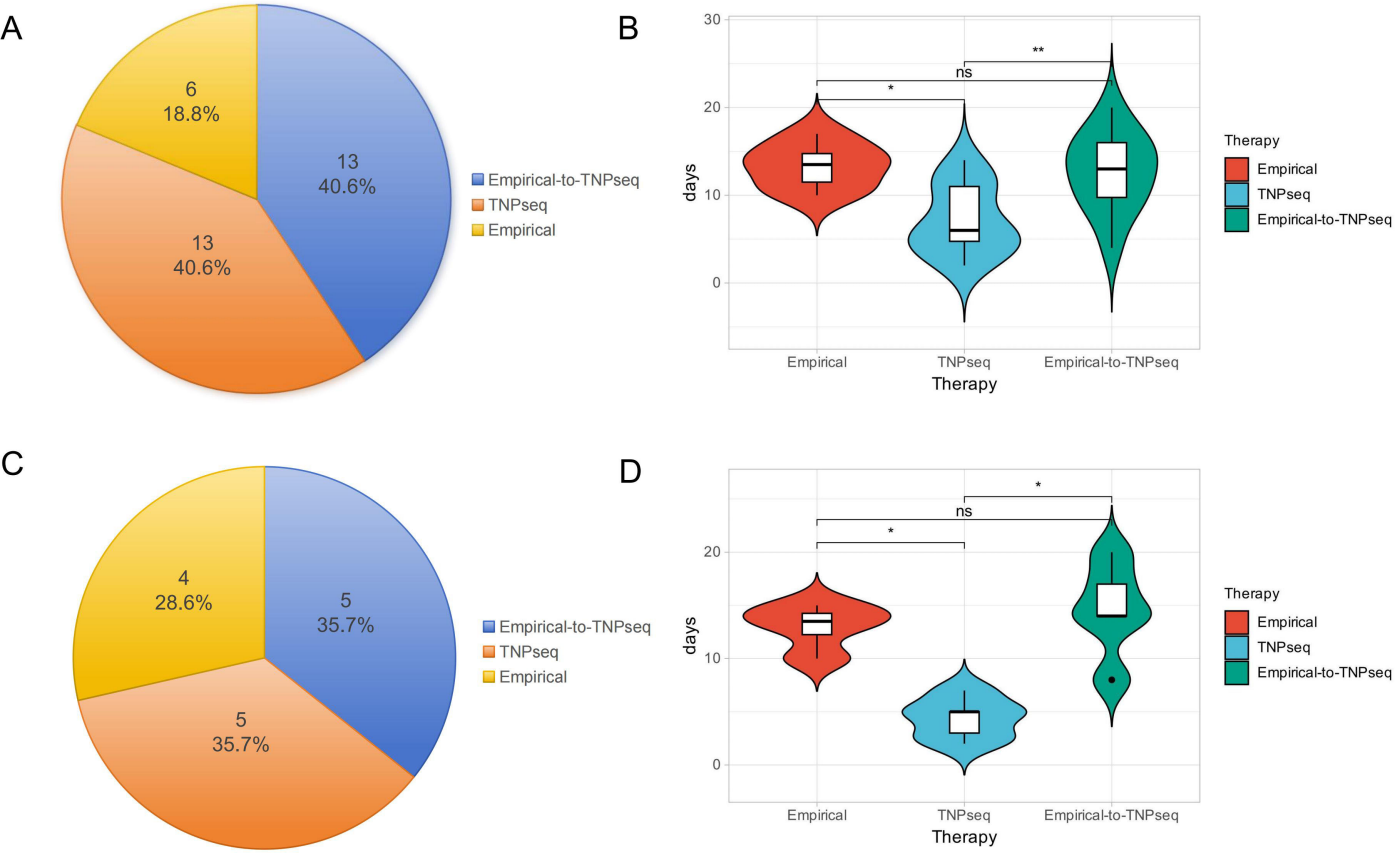
Therapeutic strategy distribution and duration in the treatment cohort. **(A)** Proportion of patients receiving three therapeutic strategies: empirical, TNPseq-guided, and empirical-to-TNPseq conversion; **(B)** Therapy duration (days) for empirical, TNPseq-guided, and empirical-to-TNPseq approaches; **(C)** Proportion of lung cancer (LC) patients managed under three treatment modalities; **(D)** Therapy cycle length (days) for LC subgroup across three regimens. *P<0.05, **P<0.01, ns, non-significant.

We evaluated clinical outcomes among 32 patients (14 with LC, 18 with BPD), comparing initial empirical antibiotic therapy (n=19) with direct TNPseq-guided therapy (n=13). Clinical improvement was ultimately achieved in all patients. However, among those who initially received empirical therapy, 68.4% (13/19) showed no improvement and required subsequent treatment adjustment based on TNPseq findings (**Figure 5A**). When directly guided by TNPseq from the outset, patients experienced a median treatment duration that was 53.8% shorter than that of the empirical-to-TNPseq therapy group (6 days vs. 13 days; P<0.01) (**Figure 5B**). For instance, a patient presenting with cough, yellow-white sputum, and chest tightness showed no improvement after 12 days of empirical cefuroxime. Subsequent TNPseq analysis identified *Stenotrophomonas maltophilia* (missed by conventional culture), and the patient improved after 7 days of targeted trimethoprim-sulfamethoxazole therapy.

Within the LC subgroup (n=14), 35.7% (5/14) required switching from empirical to TNPseq-guided therapy due to initial therapeutic failure (**Figure 5C**). We preliminarily found that direct TNPseq-guided therapy significantly shortened the median treatment duration (5 days vs. 15.5 days; P<0.05) compared to the empirical-to-TNPseq transition (**Figure 5D**). TNPseq effectively reduced unnecessary antibiotic use. For example, an elderly patient with suspected pneumonia failed initial empirical levofloxacin therapy; TNPseq testing revealed *human herpesvirus 4* without bacterial or fungal pathogens. Antibiotics were discontinued, and lung adenocarcinoma was later confirmed by biopsy. Another culture-negative patient underwent TNPseq, detecting *Haemophilus influenzae*, *human herpesvirus 4*, and the active β-lactam resistance gene *bla_TEM-1_*. Prompt targeted therapy with levofloxacin and acyclovir led to symptom resolution within 7 days, avoiding broader-spectrum antibiotics. These findings underscore TNPseq’s efficacy in optimizing infection treatment and supporting antimicrobial stewardship in patients with cancer.

## Discussion

This study demonstrates that TNPseq serves as a rapid and accurate diagnostic tool for managing pulmonary infections in lung cancer patients. By leveraging non-invasive sputum samples, TNPseq achieved comparable diagnostic sensitivity to mNGS. Furthermore, TNPseq reduced the turnaround time by 40.7% compared to mNGS (16 hours vs. 27 hours), offering a critical advantage in LC management for rapidly informing therapeutic decisions.

Unlike mNGS, which provides a broad but often noisy overview of the microbiome that can obscure clinically relevant signals and require extensive bioinformatic filtering (Govender et al., 2021), TNPseq employs targeted enrichment to amplify pathogenic sequences, thereby drastically reducing host and environmental background interference. TNPseq significantly outperformed mNGS in clinical pathogen detection (48.76% vs. 16.80%), consistent with previous findings in cerebrospinal fluid pathogen detection studies comparing targeted sequencing and mNGS methods (Chen et al., 2024).

Within the treatment cohort, we further evaluated the impact of TNPseq-guided therapy against empirical treatment. Notably, 68.4% (13/19) of patients initially receiving empirical therapy required a transition to TNPseq-guided treatment due to inadequate response. TNPseq-guided management significantly outcomes, achieving clinical improvement in all patients (100%) and reducing the median treatment duration by 53.8% compared to the empirical-to-TNPseq switch group (6 days vs. 13 days, P < 0.01). This reduction was even more pronounced in the LC subgroup specifically, where treatment duration decreased by 67% (P < 0.05). It should be noted that this is a preliminary observation from a small sample, and future larger-scale clinical validation is needed. This notable reduction in treatment duration likely stems from the complex interplay between tumor and infection in cancer patients. Tumor cells release cytokines and growth factors, impairing host anti-infective defenses, while infection-induced inflammation can drive tumor cell proliferation and metastasis (Cattaneo et al., 2020; Liu et al., 2020a). These interactions complicate the tumor microenvironment, making empirical treatments less effective.

Optimized antibiotic use is particularly crucial in cancer patients, as chemotherapy indirectly elevates the risk of antibiotic resistance in cancer patients (Sharma et al., 2025), a factor linked to poor clinical outcomes (Nanayakkara et al., 2021). TNPseq, as a sensitive nucleic acid-based diagnostic approach, effectively addresses the limitations of empirical strategies. By accurately detecting pathogens and associated resistance genes, TNPseq enables precise, targeted therapies and reduces unnecessary prolonged use of broad-spectrum antibiotics. Additionally, its capability to identify viral pathogens (e.g., *human herpesvirus 4*) also provides evidence for avoiding antibiotic overuse.

The use of sputum samples offers a safer alternative for LC patients by avoiding invasive procedures such as bronchoalveolar lavage. Although historically limited by lower microbial yields and contamination, advances in targeted enrichment technology have effectively addressed these issues (Feigelman et al., 2017). Previous validations have confirmed the non-inferior sensitivity of TNPseq in sputum samples, aligning with published nanopore detection thresholds of 102–104 copies/ml (Deng et al., 2022). Thus, sputum-based TNPseq provides dual clinical benefits: safeguarding fragile pulmonary health in oncology patients and enabling prompt and precise infection management essential for uninterrupted anticancer treatment.

Microorganisms within pulmonary tumors interact with the external environment via the respiratory tract, potentially becoming trapped in respiratory mucosa. This positions sputum as a valuable diagnostic specimen for capturing microbial signatures associated with LC (Gagliani et al., 2014). In this study, LEfSe analysis revealed significant fungal diversity differences between NDLC and BPD groups. Both mNGS and TNPseq identified *Lactobacillus* as a dominant genus in NDLC samples. Previous research indicates *Lactobacillus* supplementation can reduce pulmonary inflammation, offering potential therapeutic benefits in conditions such as asthma and COPD (Elean et al., 2023). However, each standard deviation increase in *Lactobacillus* abundance was linked to a 6% higher risk of lung cancer (HR = 1.06, 95%CI: 1.03-1.09), revealing the context-dependent nature of commensal bacteria (Vogtmann et al., 2022).

We also examined microbial profiles associated with LC therapy responses. Both sequencing methods revealed preliminary differences, with potential enrichment of *Neisseria*, *Veillonella*, and *Prevotella* in the PR/SD group compared to the PD group. *Prevotella* and *Veillonella* are typically among the most abundant genera in healthy lungs (Liu et al., 2020b). Oral commensals such as *Prevotella* and *Veillonella* have previously been shown to activate ERK and PI3K pathways, which correlate with improved LC therapy responses (Tsay et al., 2018). *Prevotella* species, capable of inducing mild inflammation, may foster respiratory immune tolerance, which could partly explain their dominance during the PR phase of treatment (Larsen et al., 2015). Our findings align with these observations, suggesting a potential role of *Prevotella* in modulating responses to LC therapy. However, these associations are exploratory and derived from a limited sample size, necessitating confirmation in larger, prospectively designed cohorts. Moreover, our TNPseq analysis suggested a potential enrichment of *Streptococcus intermedius* in PD samples. This finding may be clinically relevant, as previous studies have reported *Streptococcus intermedius* enrichment in synchronous multiple primary lung cancer patients, where it has been proposed to facilitate tumor progression by shortening cell cycles and inhibiting apoptosis (Deng et al., 2025). This observation hints at the ability of TNPseq to detect potentially pathogenic bacteria that might be less prominently captured by mNGS, though further studies are needed to validate its specificity and prognostic value.

Several limitations of this study should be acknowledged. Its retrospective, single-center design and limited sample size may lead to selection bias and unmeasured confounding. Factors including heterogeneous baseline disease severity, comorbidities, tumor treatment regimens, and pathogen types could influence treatment outcomes and microbiome associations. The lack of multivariate adjustment for these confounders limits causal interpretation regarding TNPseq’s impact on treatment duration. Furthermore, while TNPseq showed advantages in speed and specificity for pathogen detection, its scope did not include RNA viruses, which are common in respiratory infections. Future implementations should therefore expand detection to include RNA viruses. Additional limitations include single-timepoint sampling, which precludes assessment of microbiome dynamics, and the absence of host immune profiling, which hinders mechanistic insight. These factors may influence the interpretation of microbiome biomarkers and their clinical links. Consequently, future prospective, multi-center studies with larger, longitudinally sampled cohorts are needed. Employing stratified designs and multivariate adjustments will allow a more robust evaluation of TNPseq’s clinical utility and cost-effectiveness as a diagnostic strategy.

## Conclusion

TNPseq demonstrates strong potential as an effective diagnostic platform for pulmonary infections in lung cancer patients, enabling rapid pathogen detection and treatment guidance via non-invasive sputum sampling. Compared to empirical therapy, TNPseq-guided treatment achieved a high clinical improvement rate and significantly shortened antibiotic duration, thereby reducing unnecessary broad-spectrum exposure, which is particularly important for immunocompromised patients undergoing anticancer therapy. Beyond acute infection management, our exploratory analyses suggest that TNPseq can reveal differences in microbiome composition across disease groups and identified microbial features potentially associated with tumor treatment response. Collectively, these findings position TNPseq as a promising candidate for a first-line diagnostic strategy, though its implementation in this role requires validation through prospective, cost-effectiveness studies.

## Data Availability

The datasets generated and/or analyzed during the current study are available in the National Genomics Data Center repository with the reference number PRJCA045595.

## Supplementary material

Supplementary Figure 1 Study population flowchart for pulmonary infection diagnosis and treatment.

Supplementary Figure 2 Clinical diagnostic flowchart for suspected Mycoplasma pneumoniae infection based on Composite Reference Standards (CRS).

Supplementary Figure 3 Distribution of the species among bacteria, fungi, virus and other pathogens detected by TNPseq and mNGS

Supplementary Figure 4 Comparison of α-diversity indices across study groups (BPD, IDLC, NDLC) for different pathogen types. **(A–D)** show results from mNGS, and **(E–H)** show results from TNPseq. Each row presents a different α-diversity metric: Richness **(A, E)**, Simpson index **(B, F)**, ACE index **(C, G)**, and Chao1 index **(D, H)**. BPD: Benign pulmonary disease; IDLC: Initial diagnosis lung cancer; NDLC: Non-initial diagnosis lung cancer; SP: Specific pathogen.

Supplementary Figure 5 Comparison of mNGS and TNPseq for pathogen detection in PR/SD and PD cohorts. **(A)**. RACs demonstrate that mNGS achieves greater taxonomic breadth and evenness, evidenced by longer, flatter curves; **(B)**. α-diversity (via Shannon index) was calculated using mNGS-detected data for assay comparison; **(C)**. β-diversity was calculated via PCoA using mNGS-detected data for assay comparison; **(D)**. Relative abundance of each pathogen in each group, as measured by mNGS; **(E)**. RACs demonstrate that TNPseq more reliably detects dominant pathogens, as reflected by steeper curves. **(F)**. α-Diversity was calculated using TNPseq-detected data for assay comparison; **(G)**. β-Diversity was calculated using TNPseq-detected data for assay comparison; **(H)**. Relative abundance of each pathogen in each group, as measured by TNPseq. ns: non-significant. SP: Specific pathogen

Supplementary Figure 6 Comparison of α-diversity indices between treatment response groups (PR/SD vs. PD) for different pathogen types. **(A–D)** show results from mNGS, and **(E–H)** show results from TNPseq. Each row presents a different α-diversity metric: Richness **(A, E)**, Simpson index **(B, F)**, ACE index **(C, G)**, and Chao1 index **(D, H)**.
