# Supplementary figures and images for: Clinical validation and utility of targeted nanopore sequencing for rapid pathogen diagnosis and precision therapy in lung cancer patients with pulmonary infections
Source: Front Cell Infect Microbiol. 2026 Jan 12;15:1730098. doi: 10.3389/fcimb.2025.1730098 (PMC12833418; doi:10.3389/fcimb.2025.1730098)

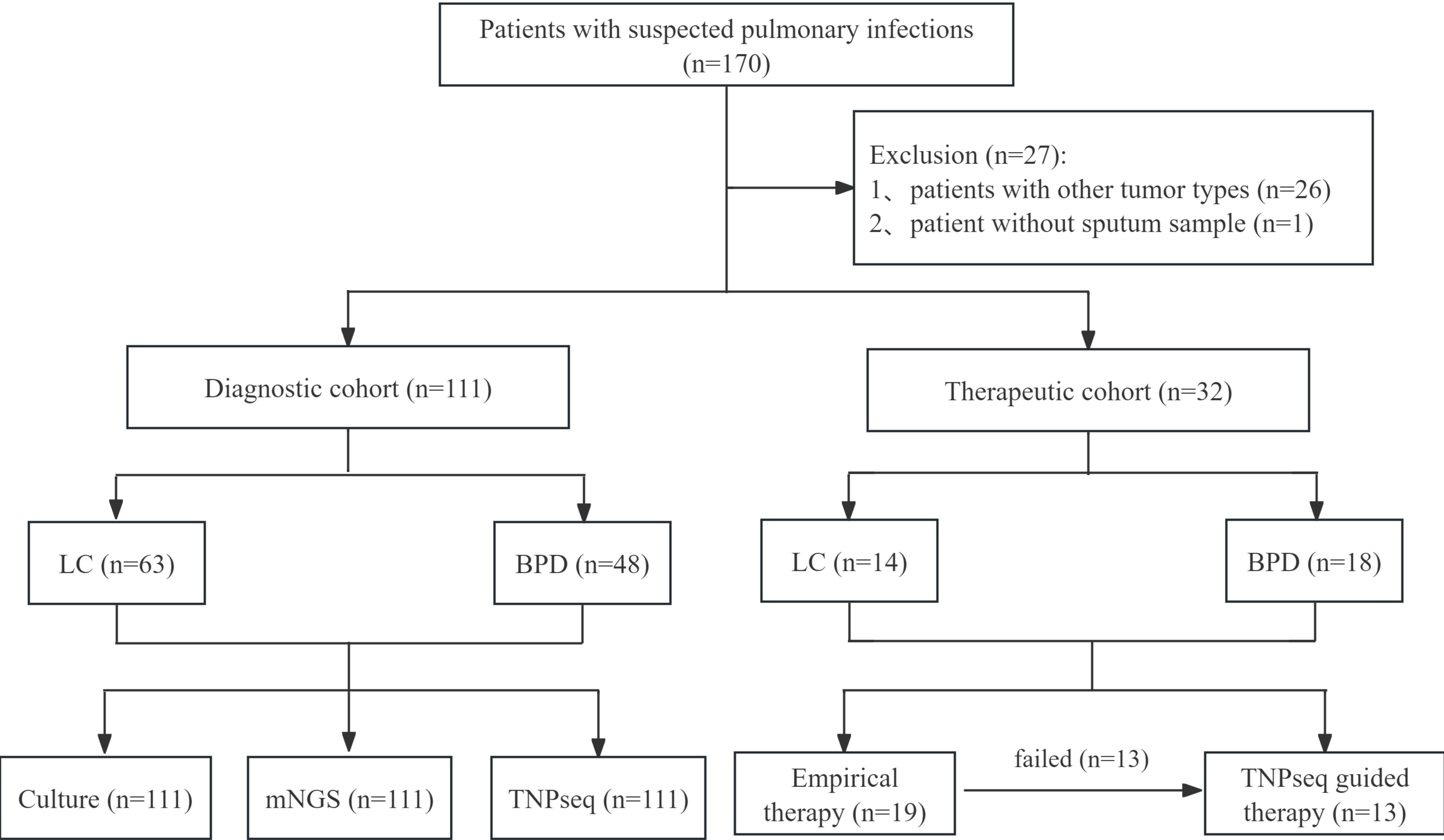

Supplement: Supplementary Figure 1 — Study population flowchart for pulmonary infection diagnosis and treatment. [file Image1.pdf]

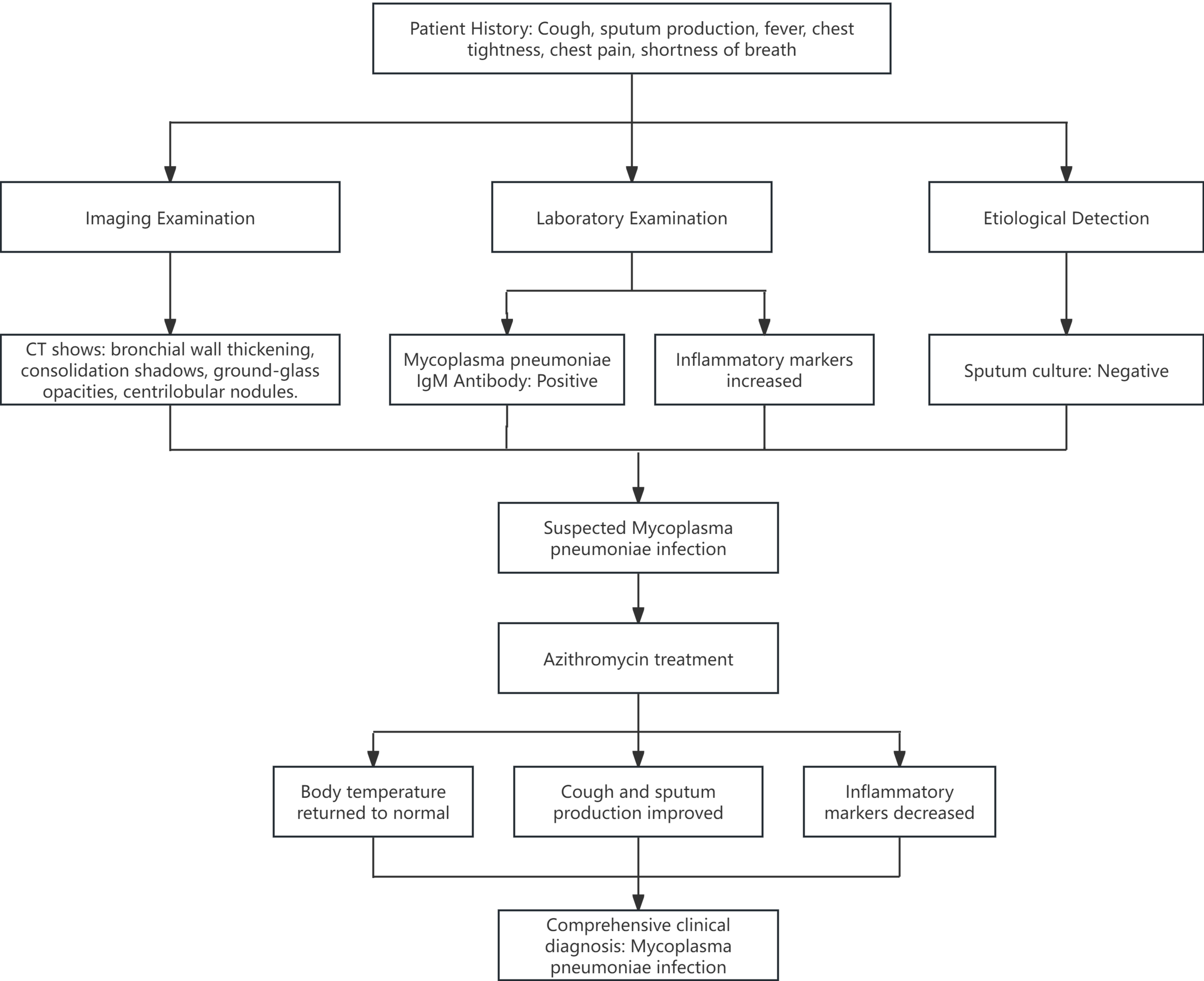

Supplement: Supplementary Figure 2 — Clinical diagnostic flowchart for suspected Mycoplasma pneumoniae infection based on Composite Reference Standards (CRS). [file Image2.pdf]

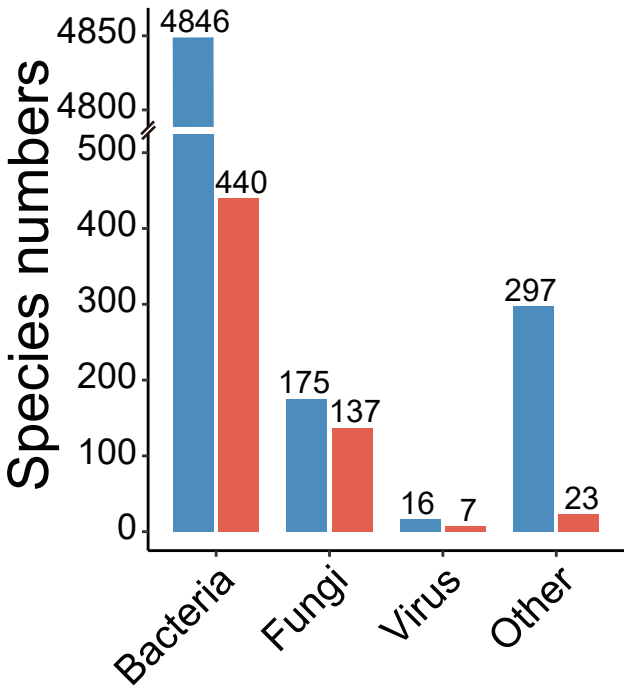

Supplement: Supplementary Figure 3 — Distribution of the species among bacteria, fungi, virus and other pathogens detected by TNPseq and mNGS [file Image3.pdf]

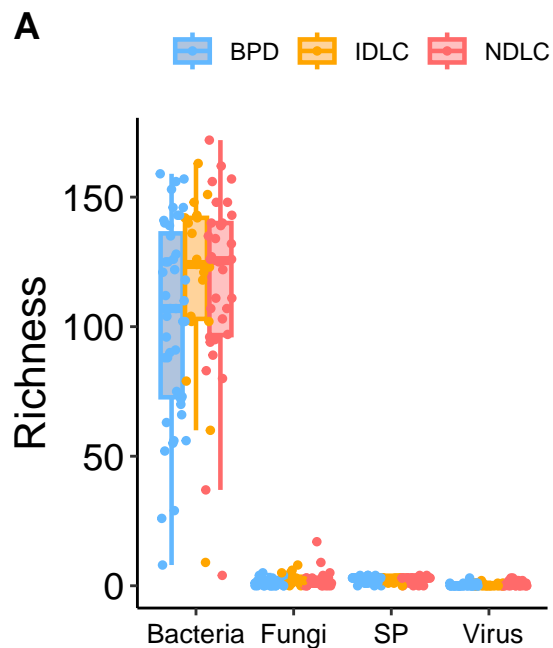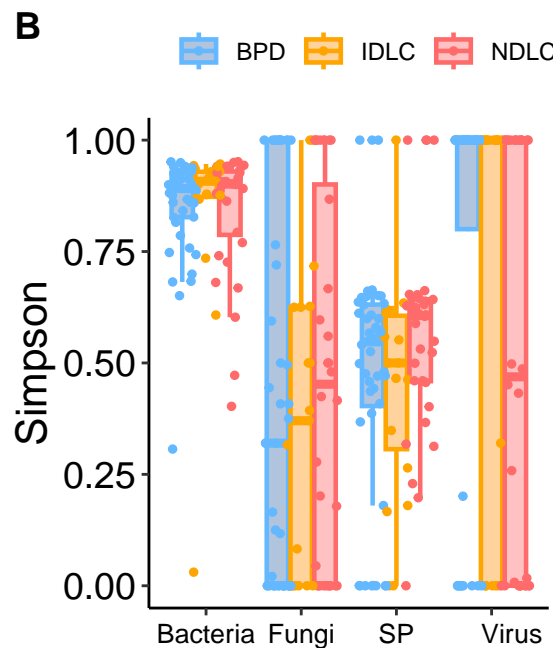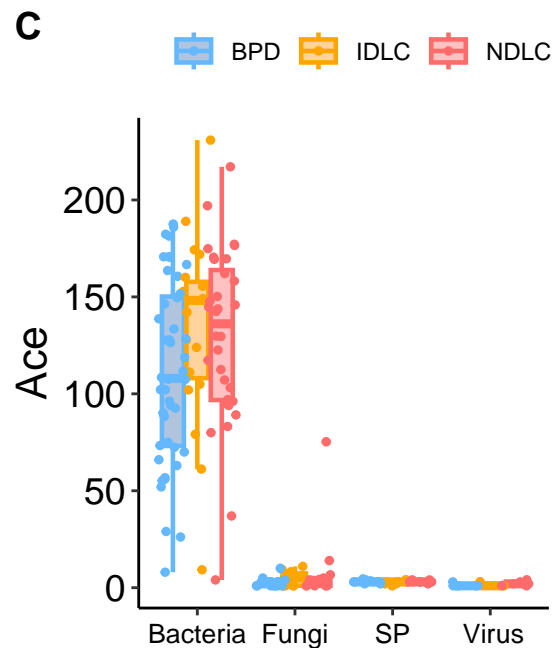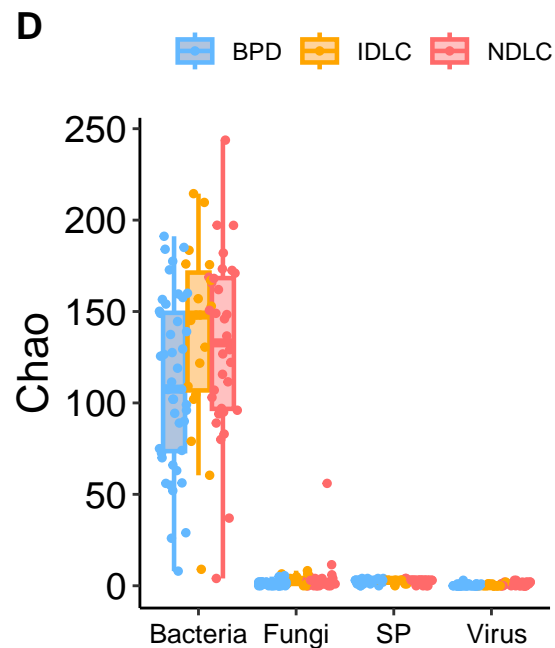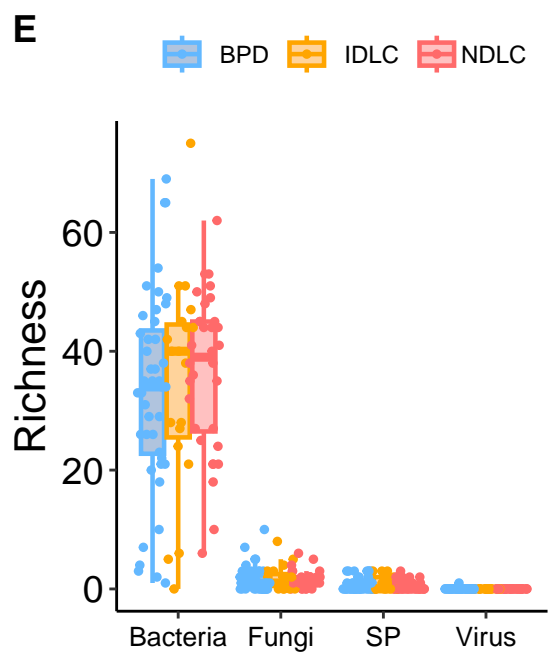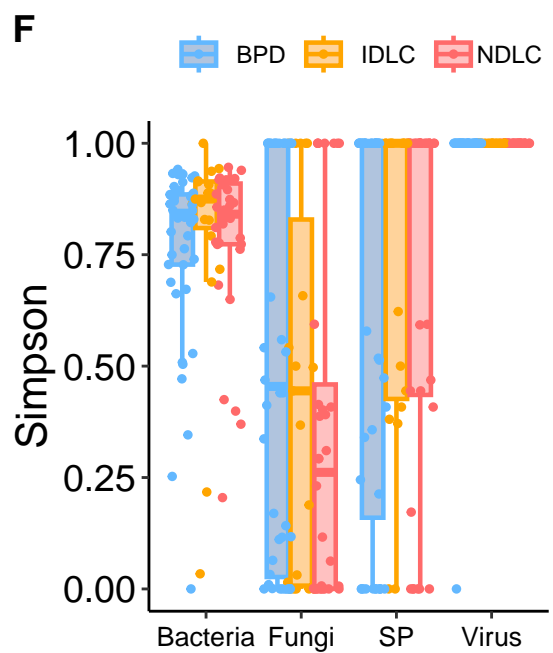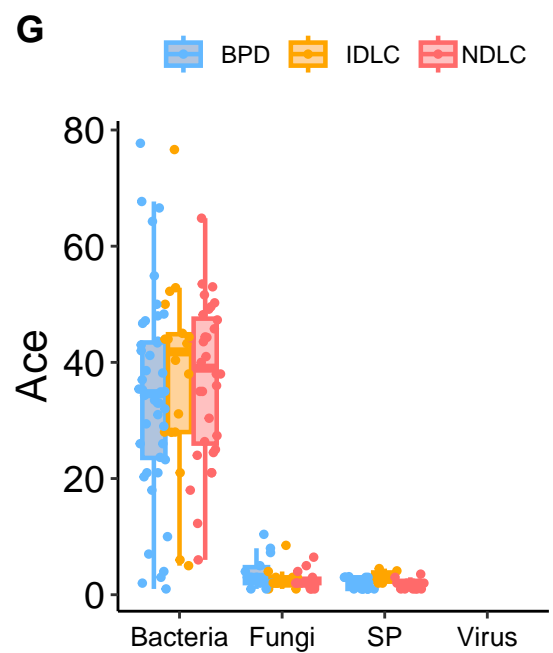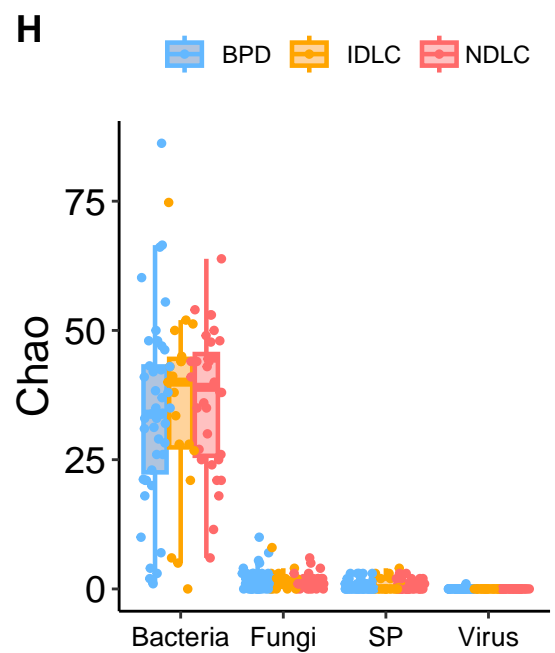

Supplement: Supplementary Figure 4 — Comparison of α-diversity indices across study groups (BPD, IDLC, NDLC) for different pathogen types. (A–D) show results from mNGS, and (E–H) show results from TNPseq. Each row presents a different α-diversity metric: Richness (A, E), Simpson index (B, F), ACE index (C, G), and Chao1 index (D, H). BPD: Benign pulmonary disease; IDLC: Initial diagnosis lung cancer; NDLC: Non-initial diagnosis lung cancer; SP: Specific pathogen. [file Image4.pdf]

Figure S3

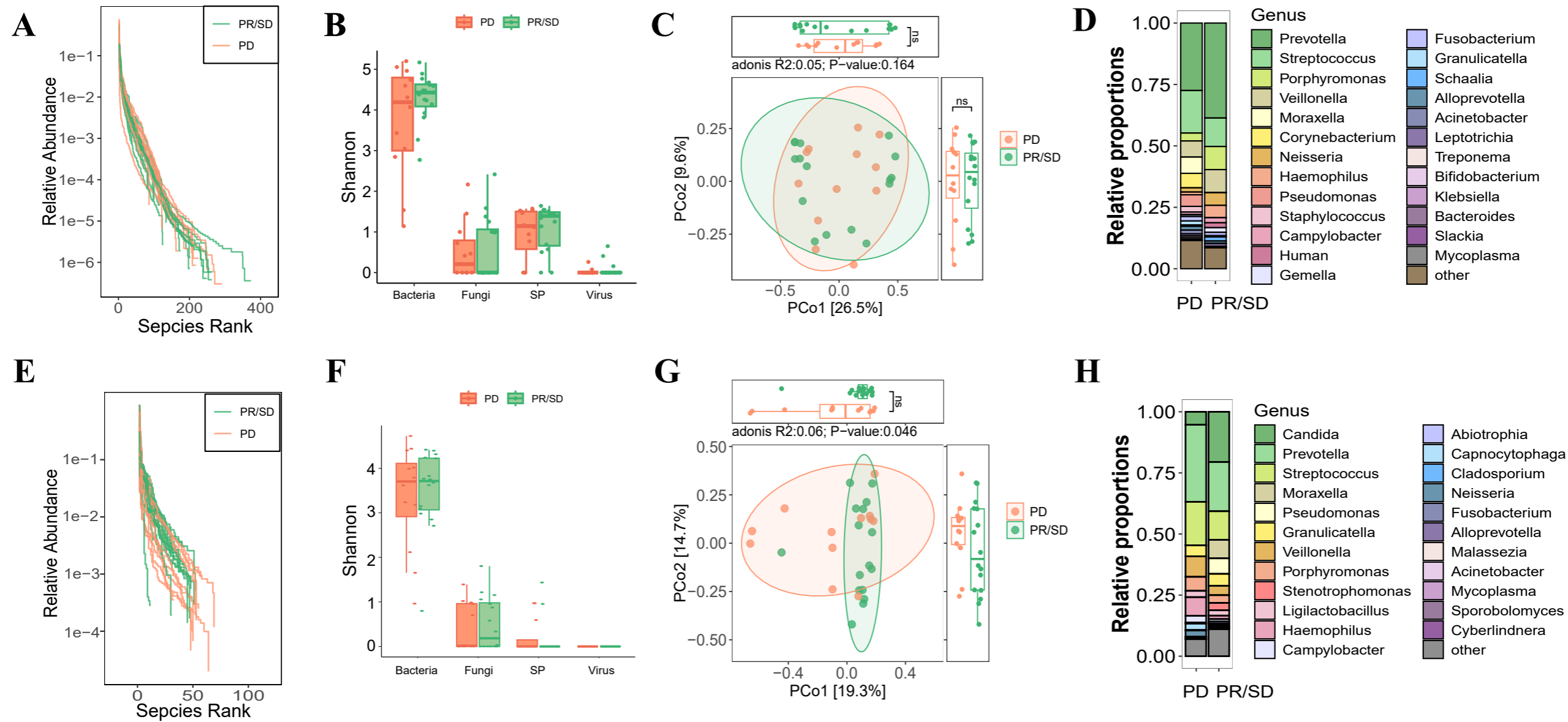

Supplement: Supplementary Figure 5 — Comparison of mNGS and TNPseq for pathogen detection in PR/SD and PD cohorts. (A). RACs demonstrate that mNGS achieves greater taxonomic breadth and evenness, evidenced by longer, flatter curves; (B). α-diversity (via Shannon index) was calculated using mNGS-detected data for assay comparison; (C). β-diversity was calculated via PCoA using mNGS-detected data for assay comparison; (D). Relative abundance of each pathogen in each group, as measured by mNGS; (E). RACs demonstrate that TNPseq more reliably detects dominant pathogens, as reflected by steeper curves. (F). α-Diversity was calculated using TNPseq-detected data for assay comparison; (G). β-Diversity was calculated using TNPseq-detected data for assay comparison; (H). Relative abundance of each pathogen in each group, as measured by TNPseq. ns: non-significant. SP: Specific pathogen [file Image5.pdf]

**A**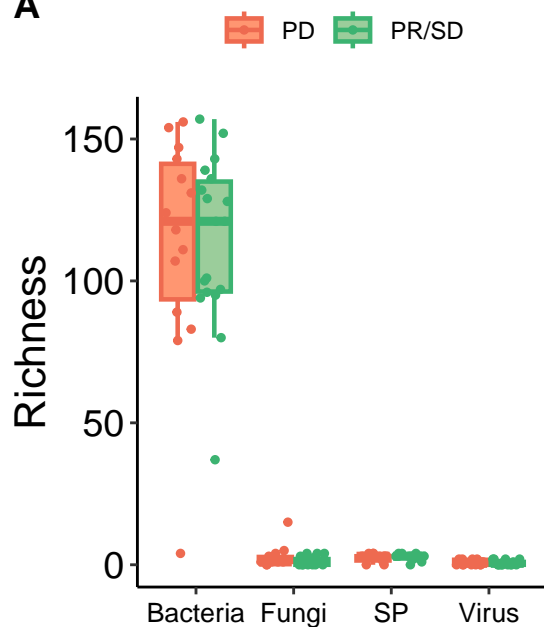**B**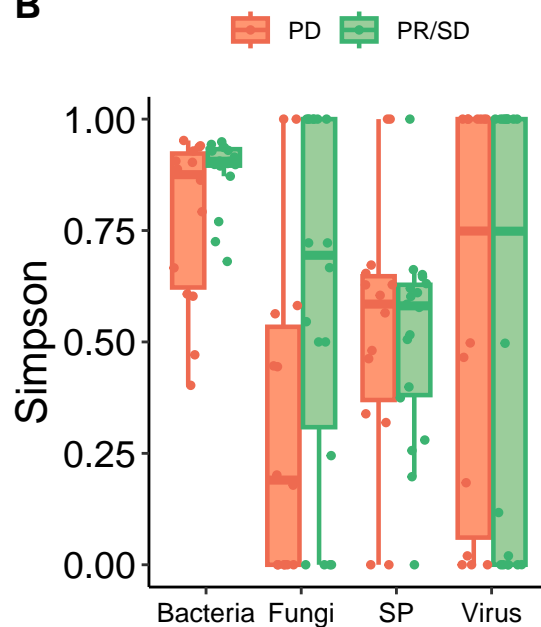**C**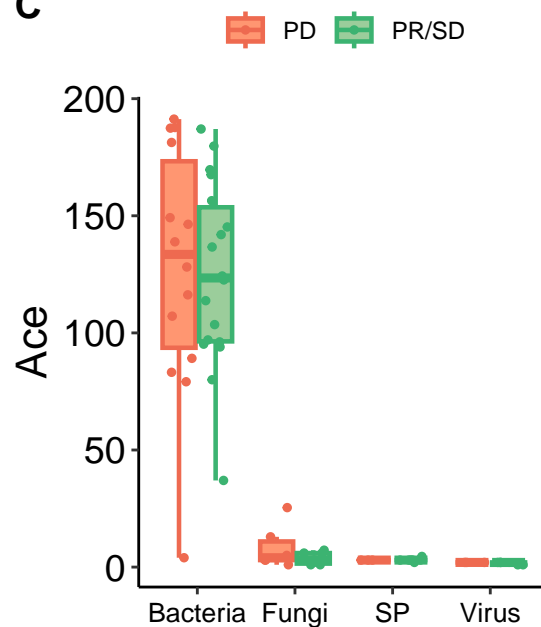**D**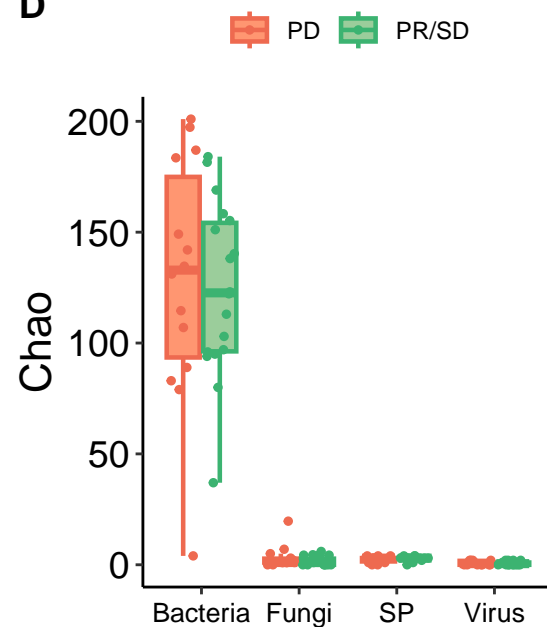**E**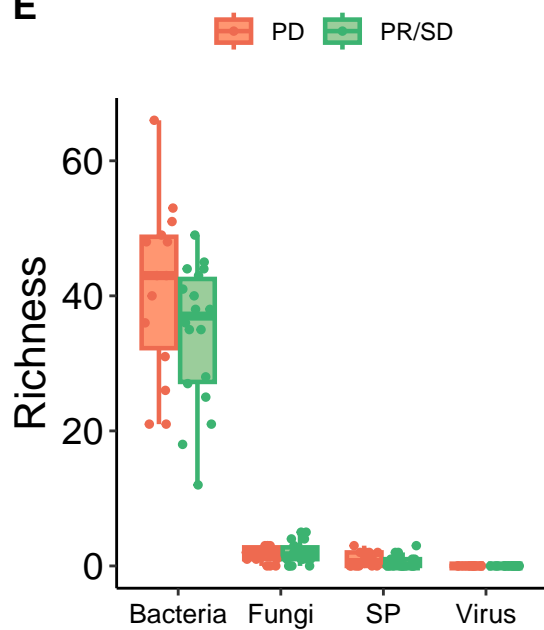**F**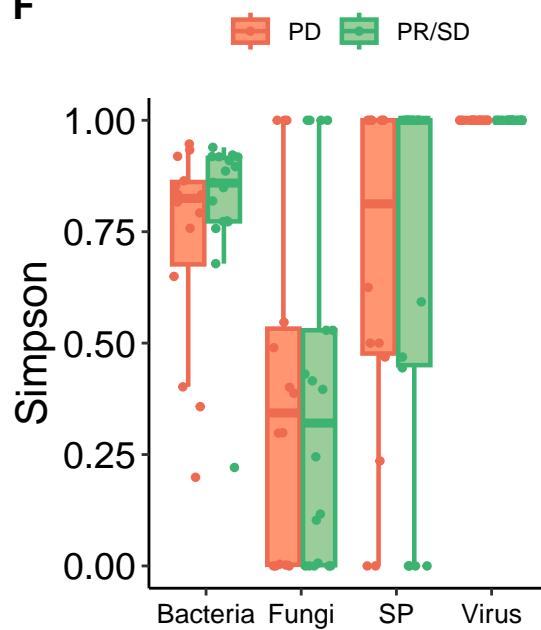**G**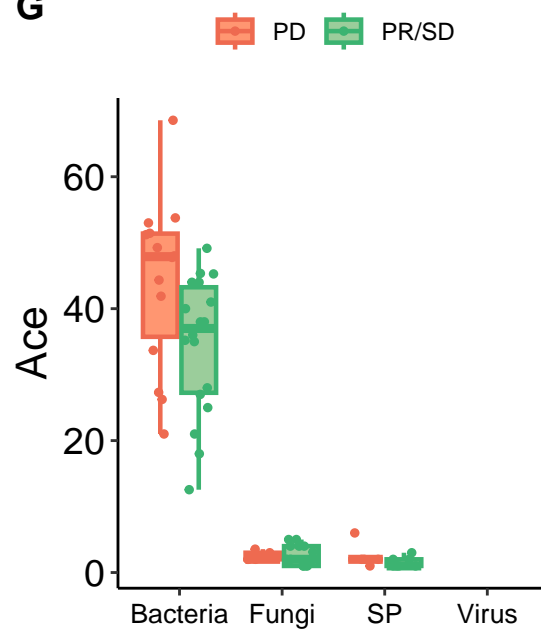**H**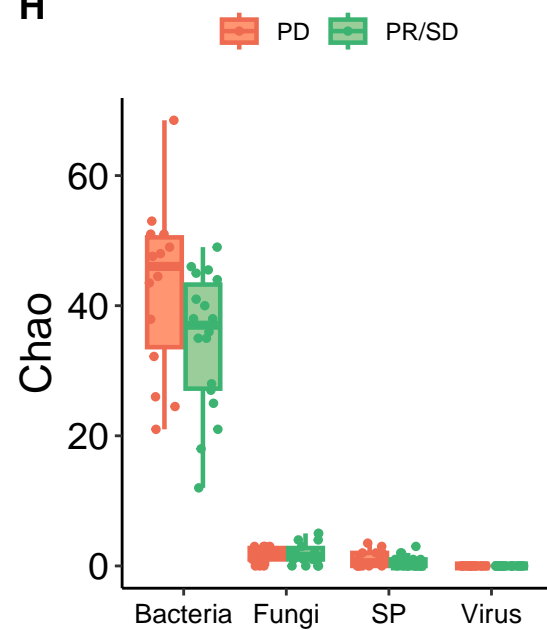

Supplement: Supplementary Figure 6 — Comparison of α-diversity indices between treatment response groups (PR/SD vs. PD) for different pathogen types. (A–D) show results from mNGS, and (E–H) show results from TNPseq. Each row presents a different α-diversity metric: Richness (A, E), Simpson index (B, F), ACE index (C, G), and Chao1 index (D, H). [file Image6.pdf]
