# Supplementary material for: Clinical validation and utility of targeted nanopore sequencing for rapid pathogen diagnosis and precision therapy in lung cancer patients with pulmonary infections
Source: Front Cell Infect Microbiol. 2026 Jan 12;15:1730098. doi: 10.3389/fcimb.2025.1730098 (PMC12833418; doi:10.3389/fcimb.2025.1730098)
Supplement: Supplementary file 7 [file DataSheet1.pdf]

Table S1 Common microorganisms in pulmonary diseases among lung cancer patients (excluding RNA viruses)

Type	Scientific Name
Bacteria	Acinetobacter baumannii
	Acinetobacter calcoaceticus
	Acinetobacter pittii
	Burkholderia cepacia
	Chlamydia psittaci
	Citrobacter freundii
	Corynebacterium striatum
	Enterobacter cloacae
	Enterococcus faecium
	Escherichia coli
	Escherichia fergusonii
	Haemophilus influenzae
	Haemophilus parainfluenzae
	Klebsiella aerogenes
	Klebsiella oxytoca
	Klebsiella pneumoniae
	Moraxella catarrhalis
	Mycobacterium avium
	Mycobacterium canariasense
	Mycobacterium intracellulare
	Mycobacterium paragordonae
	Mycobacterium phocaicum
	Mycobacterium tuberculosis
	Mycobacteroides chelonae
	Mycolicibacterium mucogenicum
	Nocardia farcinica
	Proteus mirabilis
	Pseudomonas aeruginosa
	Serratia marcescens
	Staphylococcus aureus
	Stenotrophomonas maltophilia
	Streptococcus mitis
	Streptococcus milleri group
	Streptococcus parasanguinis
	Streptococcus pneumoniae
	Streptococcus pseudopneumoniae
	Streptococcus pyogenes
	Aspergillus flavus
	Aspergillus fumigatus
	Aspergillus glaucus
	Aspergillus gracilis
	Aspergillus nidulans

Fungi	Aspergillus niger
	Candida albicans
	Candida glabrata
	Candida parapsilosis
	Candida tropicalis
	Cryptococcus neoformans
	Malassezia restricta
	Pneumocystis jirovecii
	Saccharomyces cerevisiae
	Scedosporium apiospermum
Specific pathogen	Mycoplasma pneumoniae
Viruses	Human alphaherpesvirus 1
	Human alphaherpesvirus 2
	Human betaherpesvirus 5
	Human betaherpesvirus 6
	Human betaherpesvirus 7
	Human gammaherpesvirus 4
	Human papillomavirus type 16
	Primate erythroparvovirus 1
	Torque teno virus
