# Supplementary material for: Clinical validation and utility of targeted nanopore sequencing for rapid pathogen diagnosis and precision therapy in lung cancer patients with pulmonary infections
Source: Front Cell Infect Microbiol. 2026 Jan 12;15:1730098. doi: 10.3389/fcimb.2025.1730098 (PMC12833418; doi:10.3389/fcimb.2025.1730098)
Supplement: Supplementary file 8 [file DataSheet2.pdf]

Table S2 Turnaround time per batch using mNGS and TNPseq

Steps	Turnaround time (h)	
	mNGS	TNPseq
DNA extraction	1	1
Target enrichment (multiplex PCR)	/	4
Library preparation	2	2
Quality control	0.5	0.5
Sequencing	22	7
Analysis	1.5	1.5
Total	27	16
