# Supplementary material for: Clinical validation and utility of targeted nanopore sequencing for rapid pathogen diagnosis and precision therapy in lung cancer patients with pulmonary infections
Source: Front Cell Infect Microbiol. 2026 Jan 12;15:1730098. doi: 10.3389/fcimb.2025.1730098 (PMC12833418; doi:10.3389/fcimb.2025.1730098)
Supplement: Supplementary file 9 [file DataSheet3.pdf]

Table S3 Comparison of sequencing results to culture and clinical diagnosis

Methods	Culture (+)			CRS (+)			Culture (-)CRS (-)		
		Cases	Sensitivity		Cases	Sensitivity		Cases	Percentage
mNGS	+	8	72.73%	+	11	68.75%	+	89	93.68%
	-	3	/	-	5	/	-	6	6.32%
TNPseq	+	8	72.73%	+	13	81.25%	+	91	95.79%
	-	3	/	-	3	/	-	4	4.21%
