# Supplementary material for: Clinical validation and utility of targeted nanopore sequencing for rapid pathogen diagnosis and precision therapy in lung cancer patients with pulmonary infections
Source: Front Cell Infect Microbiol. 2026 Jan 12;15:1730098. doi: 10.3389/fcimb.2025.1730098 (PMC12833418; doi:10.3389/fcimb.2025.1730098)
Supplement: Supplementary file 10 [file DataSheet4.pdf]

Table S4 Detection of Pathogens Using mNGS and TNPseq

ID	mNGS	TNPseq
1	Haemophilus hemolyticus, Haemophilus influenzae, Streptococcus pneumoniae,	Pseudomonas aeruginosa, Corynebacterium striatum
2	Human gammaherpesvirus 4, Streptococcus pneumoniae	Human gammaherpesvirus 4, Human alphaherpesvirus 1, Human
3	Pseudomonas aeruginosa, Human gammaherpesvirus 4	Pseudomonas aeruginosa, Escherichia coli, Corynebacterium striatum, Candida albicans, Human gammaherpesvirus 4
4	Acinetobacter baumannii, Escherichia coli, Human betaherpesvirus 5, Haemophilus hemolyticus, Streptococcus pneumoniae, Candida	Escherichia coli, Acinetobacter baumannii, Candida albicans, Candida tropicalis, Human betaherpesvirus 5
5	Acinetobacter baumannii, Candida albicans	Escherichia coli, Acinetobacter baumannii, Candida albicans, Human betaherpesvirus 5, Human alphaherpesvirus 1, Human
6	Pseudomonas aeruginosa	Pseudomonas aeruginosa, Klebsiella pneumoniae
7	Streptococcus pneumoniae, Haemophilus influenzae	Human betaherpesvirus 7, Streptococcus pneumoniae
8	Streptococcus pneumoniae, Haemophilus influenzae, Human gammaherpesvirus 4, Candida albicans	Haemophilus influenzae, Candida albicans, Human gammaherpesvirus 4, Human alphaherpesvirus 1
9	Haemophilus hemolyticus, Haemophilus influenzae, Streptococcus pneumoniae, Klebsiella	Klebsiella pneumoniae, Human betaherpesvirus 7
10	Pseudomonas aeruginosa	Pseudomonas aeruginosa, Aspergillus fumigatus, Human gammaherpesvirus 4
11	Haemophilus hemolyticus, Haemophilus influenzae, Streptococcus pneumoniae, Klebsiella	Haemophilus influenzae, Streptococcus pneumoniae, Acinetobacter baumannii, Human betaherpesvirus 6, Human
12	Klebsiella pneumoniae, Candida albicans, Escherichia coli,	Klebsiella pneumoniae, Candida albicans
13	Haemophilus influenzae, Streptococcus pneumoniae,	Streptococcus pneumoniae, Human betaherpesvirus 7, Human
14	Staphylococcus aureus, Streptococcus pneumoniae	Staphylococcus aureus, Human alphaherpesvirus 1, Human alphaherpesvirus 2, Human
15	Human gammaherpesvirus 4, Streptococcus pneumoniae	Pneumocystis jirovecii, Human gammaherpesvirus 4
16	Streptococcus pneumoniae, Haemophilus influenzae	Streptococcus pneumoniae, Human gammaherpesvirus 4, Human

17	Mycobacterium tuberculosis	Mycobacterium tuberculosis, Escherichia coli, Corynebacterium striatum, Human gammaherpesvirus 4
18	Mycobacterium tuberculosis	Mycobacterium tuberculosis, Corynebacterium striatum
19	Streptococcus pneumoniae, Mycobacterium tuberculosis	Candida albicans, Human gammaherpesvirus 4, Human betaherpesvirus 7, Mycobacterium
20	Streptococcus pneumoniae, Mycobacterium tuberculosis	/
21	Streptococcus pneumoniae, Candida albicans, Human gammaherpesvirus 4, Mycobacterium tuberculosis	Mycobacterium tuberculosis, Acinetobacter baumannii, Candida albicans, Human gammaherpesvirus 4,
22	Haemophilus influenzae, Streptococcus pneumoniae, Mycoplasma pneumoniae	Haemophilus influenzae, Streptococcus pneumoniae, Human betaherpesvirus 6, Human gammaherpesvirus 4, Human
23	Streptococcus pneumoniae, Acinetobacter baumannii,	Mycobacterium tuberculosis, Acinetobacter baumannii
24	Mycobacterium tuberculosis	Mycobacterium tuberculosis, Candida
25	/	Human gammaherpesvirus 4, Human betaherpesvirus 7, Streptococcus
26	Mycoplasma pneumoniae	Candida albicans, Mycoplasma
27	Streptococcus pneumoniae, Human gammaherpesvirus 4, Mycoplasma pneumoniae	Candida albicans, Human gammaherpesvirus 4
28	Human betaherpesvirus 5, Streptococcus pneumoniae, Human alphaherpesvirus 1, Aspergillus fumigatus, Human betaherpesvirus 7	Aspergillus fumigatus, Human alphaherpesvirus 1, Human betaherpesvirus 7, Human alphaherpesvirus 2
29	Candida albicans, Streptococcus pneumoniae, Human	Candida albicans, Human gammaherpesvirus 4, Human
30	Treponema denticola	Aspergillus flavus, Treponema denticola
31	Haemophilus influenzae, Haemophilus hemolyticus, Streptococcus pneumoniae, Pneumocystis jirovecii	Pneumocystis jirovecii, Human betaherpesvirus 6, Human betaherpesvirus 7, Human
32	/	Pneumocystis jirovecii, Human betaherpesvirus 6, Human gammaherpesvirus 4, Human
33	/	Pneumocystis jirovecii, Human betaherpesvirus 7
34	Streptococcus pneumoniae, Haemophilus hemolyticus, Haemophilus influenzae	Pneumocystis jirovecii, Human gammaherpesvirus 4, Human betaherpesvirus 7, Human

35	Streptococcus pneumoniae	Human betaherpesvirus 7, Human alphaherpesvirus 1, Human alphaherpesvirus 2, Human gammaherpesvirus 4, Streptococcus
36	Haemophilus influenzae, Haemophilus hemolyticus, Streptococcus	Candida albicans
37	Streptococcus pneumoniae	Pseudomonas aeruginosa, Pneumocystis jirovecii, Human betaherpesvirus 7
38	Haemophilus hemolyticus	Candida albicans, Human
39	Haemophilus influenzae, Haemophilus hemolyticus, Streptococcus pneumoniae, Human betaherpesvirus 7	Human betaherpesvirus 7, Human gammaherpesvirus 4
40	Haemophilus hemolyticus, Haemophilus influenzae, Moraxella catarrhalis, Streptococcus pneumoniae	Human betaherpesvirus 7, Human gammaherpesvirus 4, Human betaherpesvirus 6, Mycoplasma
41	Haemophilus hemolyticus, Haemophilus influenzae, Streptococcus pneumoniae,	Mycoplasma pneumoniae
42	Haemophilus influenzae, Haemophilus hemolyticus, Streptococcus	/
43	Streptococcus pneumoniae, Haemophilus hemolyticus, Haemophilus influenzae	Pseudomonas aeruginosa, Streptococcus pneumoniae, Human alphaherpesvirus 1, Human alphaherpesvirus 2, Human gammaherpesvirus 4, Human
44	Streptococcus pneumoniae, Candida albicans	Pseudomonas aeruginosa, Escherichia coli, Candida albicans, Human gammaherpesvirus 4, Human
45	Streptococcus pneumoniae, Human gammaherpesvirus 4	Escherichia coli, Human gammaherpesvirus 4, Human betaherpesvirus 7, Human
46	Streptococcus pneumoniae, Haemophilus influenzae, Haemophilus hemolyticus, Acinetobacter baumannii, Human gammaherpesvirus 4	Human gammaherpesvirus 4, Human betaherpesvirus 7
47	Streptococcus pneumoniae	Human gammaherpesvirus 4, Streptococcus pneumoniae
48	Streptococcus pneumoniae	Human gammaherpesvirus 4, Human betaherpesvirus 7, Human alphaherpesvirus 1, Human alphaherpesvirus 2, Human
49	Haemophilus hemolyticus, Haemophilus influenzae,	Streptococcus pneumoniae
50	Haemophilus influenzae, Haemophilus hemolyticus, Human	Human gammaherpesvirus 4

51	Streptococcus pneumoniae	/
52	Candida albicans, Human gammaherpesvirus 4	/
53	Mycoplasma pneumoniae	Stenotrophomonas maltophilia, Klebsiella pneumoniae, Mycoplasma
54	Mycoplasma pneumoniae, Streptococcus pneumoniae	Stenotrophomonas maltophilia, Mycoplasma pneumoniae
55	Streptococcus pneumoniae	Streptococcus pneumoniae, Mycoplasma pneumoniae
56	Treponema denticola, Streptococcus pneumoniae, Haemophilus influenzae, Haemophilus hemolyticus, Aspergillus	Haemophilus influenzae, Human betaherpesvirus 7, Human betaherpesvirus 6
57	Haemophilus hemolyticus, Haemophilus influenzae,	Primate erythroparvovirus 1, Streptococcus pneumoniae
58	Haemophilus hemolyticus, Haemophilus influenzae, Streptococcus pneumoniae,	Human betaherpesvirus 7, Haemophilus hemolyticus
59	Haemophilus influenzae, Haemophilus hemolyticus, Streptococcus pneumoniae	Haemophilus influenzae, Candida albicans, Human betaherpesvirus 7, Human betaherpesvirus 6
60	Haemophilus influenzae, Treponema denticola, Streptococcus pneumoniae	Human betaherpesvirus 7, Streptococcus pneumoniae
61	Haemophilus influenzae, Haemophilus hemolyticus, Streptococcus	Haemophilus influenzae
62	Staphylococcus aureus	Staphylococcus aureus, Streptococcus pneumoniae, Human gammaherpesvirus
63	Haemophilus influenzae, Haemophilus hemolyticus, Streptococcus pneumoniae, Aspergillus flavus	Human betaherpesvirus 7, Human gammaherpesvirus 4, Human betaherpesvirus 6
64	Klebsiella pneumoniae, Human gammaherpesvirus 4	Streptococcus pneumoniae, Haemophilus influenzae, Klebsiella pneumoniae, Human gammaherpesvirus
65	Streptococcus pneumoniae, Haemophilus hemolyticus, Haemophilus influenzae, Human	Human gammaherpesvirus 4, Human betaherpesvirus 7, Human betaherpesvirus 6, Streptococcus
66	Haemophilus influenzae, Haemophilus hemolyticus, Streptococcus pneumoniae, Escherichia coli, Staphylococcus aureus, Human	Staphylococcus aureus, Haemophilus influenzae, Escherichia coli, Candida albicans, Human gammaherpesvirus 4, Human betaherpesvirus 6, Human
67	Haemophilus influenzae, Human gammaherpesvirus 4, Haemophilus hemolyticus, Streptococcus pneumoniae	Haemophilus influenzae, Klebsiella pneumoniae, Human gammaherpesvirus 4, Human betaherpesvirus 7, Human betaherpesvirus 5, Human

68	Haemophilus influenzae, Haemophilus hemolyticus, Streptococcus pneumoniae, Escherichia coli, Human gammaherpesvirus 4, Human	Staphylococcus aureus, Corynebacterium striatum, Escherichia coli, Human gammaherpesvirus 4, Human betaherpesvirus 7
69	Haemophilus hemolyticus, Haemophilus influenzae, Streptococcus pneumoniae, Staphylococcus aureus, Human	Staphylococcus aureus, Human betaherpesvirus 6, Human betaherpesvirus 7, Human betaherpesvirus 5
70	Streptococcus pneumoniae, Acinetobacter baumannii	Acinetobacter baumannii, Human alphaherpesvirus 1, Human gammaherpesvirus 4, Human alphaherpesvirus 2, Human
71	Human gammaherpesvirus 4, Malassezia restricta	Human gammaherpesvirus 4, Human betaherpesvirus 7
72	Moraxella catarrhalis	Mycobacterium tuberculosis, Moraxella catarrhalis, Human gammaherpesvirus 4
73	Staphylococcus aureus, Streptococcus pneumoniae, Haemophilus hemolyticus, Haemophilus influenzae	Staphylococcus aureus, Human betaherpesvirus 7
74	Streptococcus pneumoniae, Moraxella catarrhalis, Haemophilus influenzae, Haemophilus hemolyticus, Acinetobacter baumannii	Acinetobacter baumannii, Haemophilus influenzae, Human betaherpesvirus 7
75	Streptococcus pneumoniae	Escherichia coli, Streptococcus pneumoniae, Staphylococcus aureus
76	/	/
77	Haemophilus influenzae	Haemophilus influenzae, Human betaherpesvirus 6, Human
78	Streptococcus pneumoniae, Haemophilus influenzae	Haemophilus influenzae, Human betaherpesvirus 6, Human
79	Moraxella catarrhalis, Haemophilus influenzae, Streptococcus pneumoniae	Moraxella catarrhalis, Streptococcus pneumoniae
80	Haemophilus influenzae, Streptococcus pneumoniae	Haemophilus influenzae
81	Haemophilus hemolyticus, Haemophilus influenzae	Candida albicans, Human betaherpesvirus 7, Haemophilus
82	Streptococcus pneumoniae, Haemophilus influenzae, Human gammaherpesvirus 4, Pneumocystis	Stenotrophomonas maltophilia, Pneumocystis jirovecii, Aspergillus fumigatus, Human gammaherpesvirus 4
83	Streptococcus pneumoniae, Haemophilus hemolyticus,	Streptococcus pneumoniae, Candida albicans, Human gammaherpesvirus 4
84	Haemophilus influenzae, Haemophilus hemolyticus, Streptococcus pneumoniae, Candida albicans,	Aspergillus fumigatus

85	Staphylococcus aureus, Aspergillus niger, Aspergillus flavus, Candida	Candida tropicalis, Human betaherpesvirus 5, Human
86	Haemophilus influenzae, Haemophilus hemolyticus	Haemophilus influenzae, Candida albicans, Human gammaherpesvirus 4, Human betaherpesvirus 7
87	Streptococcus pneumoniae, Aspergillus flavus, Malassezia restricta	Human betaherpesvirus 7, Human betaherpesvirus 6, Enterobacter cloacae, Malassezia restricta
88	Aspergillus flavus, Candida albicans	Pseudomonas aeruginosa, Candida albicans, Aspergillus flavus, Human
89	Haemophilus influenzae, Streptococcus pneumoniae	Haemophilus influenzae, Aspergillus fumigatus, Pneumocystis jirovecii, Human gammaherpesvirus 4, Human betaherpesvirus 7, Human
90	Streptococcus pneumoniae, Staphylococcus aureus, Human	Staphylococcus aureus, Aspergillus niger, Human betaherpesvirus 5
91	Streptococcus pneumoniae, Haemophilus influenzae, Candida albicans, Human gammaherpesvirus 4	Candida albicans, Pneumocystis jirovecii, Human gammaherpesvirus 4, Human alphaherpesvirus 1
92	Streptococcus pneumoniae, Candida tropicalis, Human betaherpesvirus 5, Staphylococcus aureus	Candida tropicalis, Human betaherpesvirus 7, Human betaherpesvirus 5
93	Candida albicans, Streptococcus pneumoniae, Human gammaherpesvirus 4, Haemophilus	Candida albicans, Human gammaherpesvirus 4, Human betaherpesvirus 7
94	Streptococcus pneumoniae, Haemophilus influenzae, Haemophilus hemolyticus, Candida albicans	Candida albicans, Human betaherpesvirus 6, Human betaherpesvirus 7
95	Streptococcus pneumoniae, Candida albicans	Mycobacterium tuberculosis, Candida albicans, Streptococcus pneumoniae
96	Streptococcus pneumoniae, Candida albicans	Candida albicans, Human gammaherpesvirus 4, Human betaherpesvirus 7, Human
97	Haemophilus influenzae, Haemophilus hemolyticus, Streptococcus pneumoniae, Candida tropicalis	Candida tropicalis
98	Candida parapsilosis	Candida parapsilosis, Human gammaherpesvirus 4, Human betaherpesvirus 7, Human
99	/	Pneumocystis jirovecii, Human betaherpesvirus 7, Human gammaherpesvirus 4, Human
100	Haemophilus hemolyticus, Haemophilus influenzae, Human	Human gammaherpesvirus 4, Human betaherpesvirus 7, Human

101	Streptococcus pneumoniae, Haemophilus influenzae, Haemophilus hemolyticus, Human	Human gammaherpesvirus 4, Human betaherpesvirus 7, Human betaherpesvirus 6
102	Streptococcus pneumoniae, Human gammaherpesvirus 4, Candida albicans	Candida albicans, Human gammaherpesvirus 4
103	Streptococcus pneumoniae, Human gammaherpesvirus 4	Human gammaherpesvirus 4, Human alphaherpesvirus 1, Human alphaherpesvirus 2, Human betaherpesvirus 6, Human
104	Streptococcus pneumoniae, Haemophilus hemolyticus, Haemophilus influenzae, Human	Escherichia coli, Human gammaherpesvirus 4, Human betaherpesvirus 6, Human
105	Human gammaherpesvirus 4, Haemophilus hemolyticus, Haemophilus influenzae,	Human gammaherpesvirus 4, Human betaherpesvirus 7, Human betaherpesvirus 6
106	Streptococcus pneumoniae, Haemophilus influenzae, Haemophilus hemolyticus, Escherichia coli, Human gammaherpesvirus 4, Aspergillus	Escherichia coli, Human gammaherpesvirus 4, Human betaherpesvirus 7
107	Streptococcus pneumoniae, Haemophilus influenzae, Human gammaherpesvirus 4, Haemophilus	Human gammaherpesvirus 4, Human alphaherpesvirus 1, Human alphaherpesvirus 2
108	Streptococcus pneumoniae, Haemophilus hemolyticus,	Human gammaherpesvirus 4, Human betaherpesvirus 7, Streptococcus
109	Streptococcus pneumoniae, Human gammaherpesvirus 4	Human gammaherpesvirus 4, Human betaherpesvirus 7
110	/	Haemophilus influenzae
111	/	Human betaherpesvirus 7, Human betaherpesvirus 6
